# Supplementary material for: Estimating rates of treatment delay for malaria fevers among children in Sub-Saharan Africa 2006–2022
Source: Nat Commun. 2025 Oct 29;16:9534. doi: 10.1038/s41467-025-64584-8 (PMC12572339; doi:10.1038/s41467-025-64584-8)
Supplement: Supplementary file 1 — Supplementary Information [file 41467_2025_64584_MOESM1_ESM.pdf]

SI for:

Estimating rates of treatment delay for malaria fevers among children in Sub-Saharan Africa 2006-2022

Jailos Lubinda[1,2]†, Susan F. Rumisha[1,3,4,5]†, Paulina Dzianach[1], Michael McPhail[1], Adam Saddler[1], Annie Browne[1], Francesca Sanna[1], Yalemzewod Gelaw[1,3], Paul Castle[1], Juniper B. Kiss[1], Joseph Harris[1], Jennifer A. Rozier[1], Camilo Vargas[1], Punam Amratia[1,4], Tasmin L. Symons[1,3], Ewan Cameron[1,3], Peter W. Gething[1,3] ‡, Daniel J. Weiss [1,3] ‡

[1]The Kids Institute, Perth Children's Hospital, Nedlands, Western Australia.
[2]Macha Research Trust, Choma, Zambia.
[3]Curtin University, Bentley, Western Australia.
[4]Ifakara Health Institute, Dar es Salaam, Tanzania.
[5]National Institute for Medical Research, Dar es Salaam, Tanzania.

† These authors contributed equally

Table of Contents

1. Introduction 3

2. Supplementary Methods 3

2.2 Data Sources 3

2.3 Surveys included or excluded from the final models 4

2.3.1 Summaries of surveys available and included in the modelling 4

2.3.2 Surveys excluded from the final models 9

2.4 Data extraction process 10

2.4.1 Countries and WHO Africa subregions 12

2.5 Covariates 13

2.6 Distribution of fraction of treatment delay in reported estimates from pooled surveys 16

2.7 Model Fitting, Scaling and Control Parameters 18

2.8 Validation and Accuracy 19

3. Supplementary Results 22

Periodic Temporal Trends between 2006-2010, 2011-2015, 2016-2022 26

1. Introduction

This study modelled delayed antimalarial treatment of febrile cases of malaria in malaria-endemic countries in Africa. The study highlights the patterns of treatment delay in 46 different countries and subregions in Africa and their trends of the fraction of treatment delay between 2006 and 2022. This Supplementary File contains additional materials and information about input data processing, curation, data inclusion criteria, and additional findings.

2. Supplementary Methods

2.2 Data Sources

The input data for this analysis utilised information on the fraction of delayed treatment of febrile cases of malaria in children under 5 years old. The data was collected from 177 publicly available nationally representative population-based demographic surveys conducted in 40 current malaria-endemic countries between 2006 and 2022. Figure S1 shows each country, survey type or source and the weighted sample sizes. The surveys are mostly run using standardized sampling methods that are generally representative of the national, residence (urban-rural), and regional (subnational administrative) levels. The samples are based on stratified two-stage cluster designs of enumeration areas. Variables of interest extracted included those from additional questions measuring household wealth, residence, child age and caregiver education level.

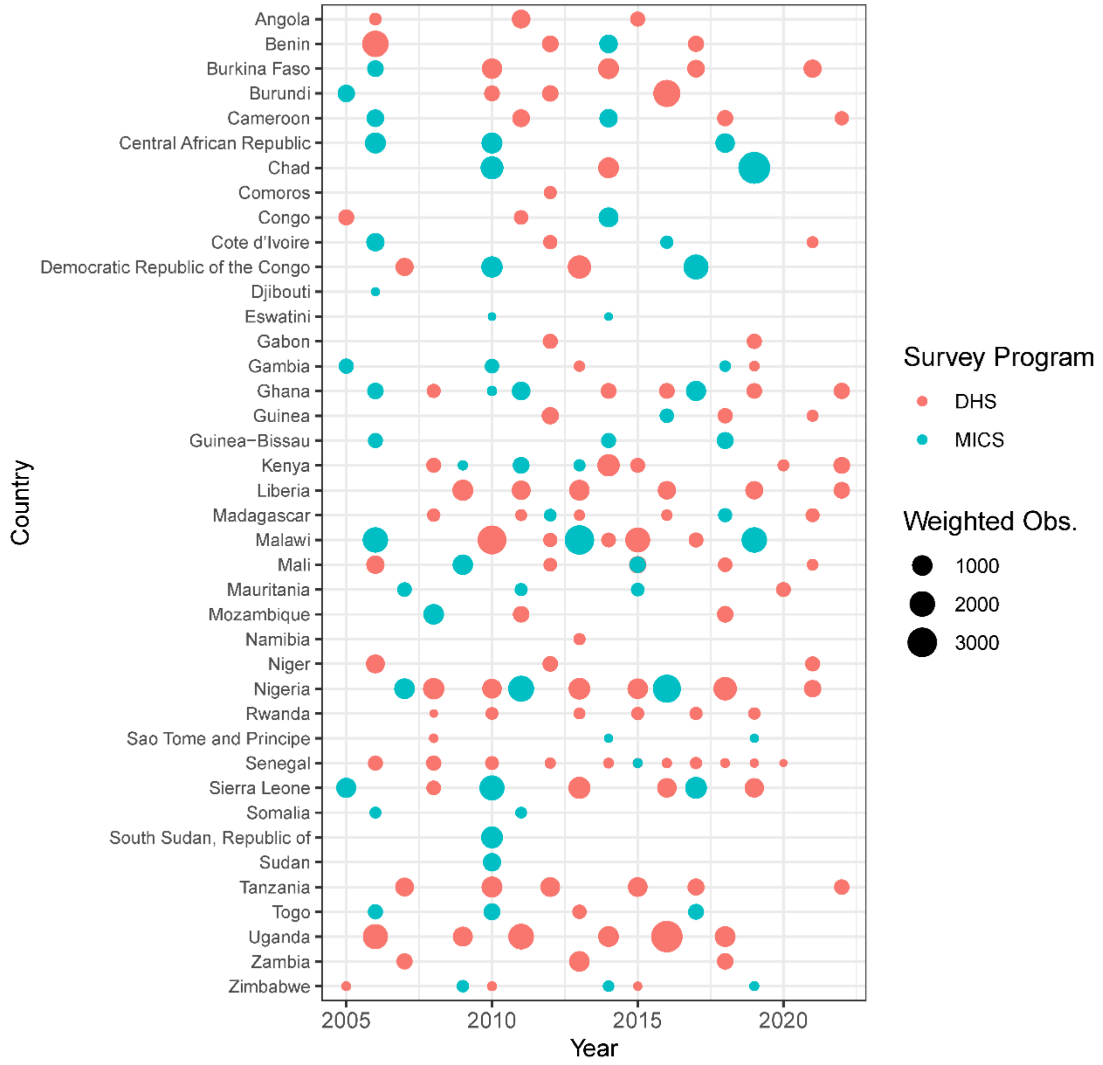

Figure S1: Data availability and summary of surveys and source
The orange dots represent data from Demographic Health Surveys (DHS), while the blue one's data from Multiple Indicator Cluster surveys (MICS). The figure shows survey type, size and year of data collection.

2.3 Surveys included or excluded from the final models

The full details, including Survey ID, type of survey, program responsible or data source and the weighted and unweighted sample sizes of each survey included in the analysis and final models, have been summarised in (Tables S1 and S2).

2.3.1 Summaries of surveys available and included in the modelling

177 surveys had questions that collected information on the occurrence of care/treatment for fever from all caregivers of children younger than 5 years and included additional specific measures of the malaria infection status among children in sampled households.

Table S1: Surveys included in the study and the number of observations

Country	Year	Program/ Source	Survey Name	Survey ID	Unweighted No. of Children	Weighted No. of Children
Angola	2006	DHS	AO2006MIS	282	141	140
Angola	2011	DHS	AO2011MIS	395	721	736
Angola	2015	DHS	AO2015DHS	477	360	317
Benin	2006	DHS	BJ2006DHS	289	2192	2194
Benin	2012	DHS	BJ2012DHS	420	473	486
Benin	2014	MICS	BEN2014MICS	271	737	724
Benin	2017	DHS	BJ2017DHS	491	429	434
Burkina Faso	2006	MICS	BFA2006MICS	166	449	484
Burkina Faso	2010	DHS	BF2010DHS	329	1036	982
Burkina Faso	2014	DHS	BF2014MIS	481	1141	1074
Burkina Faso	2017	DHS	BF2017MIS	526	636	591
Burkina Faso	2021	DHS	BF2021DHS	562	681	660
Burundi	2005	MICS	BDI2005MICS	132	550	584
Burundi	2010	DHS	BU2010DHS	346	358	389
Burundi	2012	DHS	BU2012MIS	446	409	457
Burundi	2016	DHS	BU2016DHS	463	2071	2365
Cameroon	2006	MICS	CMR2006MICS	165	646	605
Cameroon	2011	DHS	CM2011DHS	337	662	622
Cameroon	2014	MICS	CMR2014MICS	270	740	696
Cameroon	2018	DHS	CM2018DHS	511	387	424
Cameroon	2022	DHS	CM2022MIS	563	222	245
Central African Republic	2006	MICS	CAF2006MICS	164	976	1081
Central African Republic	2010	MICS	CAF2010MICS	199	1028	1095
Central African Republic	2018	MICS	CAF2018MICS	315	844	856
Chad	2010	MICS	TCD2010MICS	198	1412	1435
Chad	2014	DHS	TD2014DHS	465	902	1073
Chad	2019	MICS	TCD2019MICS	328	3202	3703
Comoros	2012	DHS	KM2012DHS	443	153	170
Congo	2005	DHS	CG2005DHS	267	363	408
Congo	2011	DHS	CG2011DHS	388	379	275
Congo	2014	MICS	COG2014MICS	272	797	925
Cote d'Ivoire	2006	MICS	CIV2006MICS	162	692	692
Cote d'Ivoire	2012	DHS	CI2012DHS	311	223	256
Cote d'Ivoire	2016	MICS	CIV2016MICS	287	178	177
Cote d'Ivoire	2021	DHS	CI2021DHS	559	115	104
Democratic Republic of the Congo	2007	DHS	CD2007DHS	239	636	664
Democratic Republic of the Congo	2010	MICS	COD2010MICS	197	1179	1147
Democratic Republic of the Congo	2013	DHS	CD2013DHS	421	1580	1521

Democratic Republic of the Congo	2017	MICS	COD2017MICS	297	1674	1873
Gabon	2012	DHS	GA2012DHS	402	306	326
Gabon	2019	DHS	GA2019DHS	546	326	337
Gambia	2005	MICS	GMB2005MICS	142	328	334
Gambia	2010	MICS	GMB2010MICS	195	247	277
Gambia	2013	DHS	GM2013DHS	425	69	74
Gambia	2018	MICS	GMB2018MICS	308	89	85
Gambia	2019	DHS	GM2019DHS	555	48	43
Ghana	2006	MICS	GHA2006MICS	160	483	468
Ghana	2008	DHS	GH2008DHS	301	231	241
Ghana	2010	MICS	2010MICS	203	30	31
Ghana	2011	MICS	GHA2011MICS	220	876	727
Ghana	2014	DHS	GH2014DHS	437	447	385
Ghana	2016	DHS	GH2016MIS	516	378	387
Ghana	2017	MICS	GHA2017MICS	296	964	969
Ghana	2019	DHS	GH2019MIS	557	428	372
Ghana	2022	DHS	GH2022DHS	598	536	438
Guinea	2012	DHS	GN2012DHS	391	539	544
Guinea	2016	MICS	GIN2016MICS	286	258	266
Guinea	2018	DHS	GN2018DHS	539	318	336
Guinea	2021	DHS	GN2021MIS	571	104	105
Guinea-Bissau	2006	MICS	GNB2006MICS	159	317	318
Guinea-Bissau	2014	MICS	GNB2014MICS	265	274	318
Guinea-Bissau	2018	MICS	GNB2018MICS	314	463	521
Kenya	2008	DHS	KE2008DHS	300	325	312
Kenya	2009	MICS	2009MICS	180	36	35
Kenya	2011	MICS	2011MICS	215	498	492
Kenya	2013	MICS	2013MICS	249	52	55
Kenya	2013	MICS	2013MICS	251	90	99
Kenya	2013	MICS	2013MICS	250	107	114
Kenya	2014	DHS	KE2014DHS	451	1354	1336
Kenya	2015	DHS	KE2015MIS	493	329	304
Kenya	2020	DHS	KE2020MIS	579	165	101
Kenya	2022	DHS	KE2022DHS	566	543	497
Liberia	2009	DHS	LB2009MIS	330	970	1066
Liberia	2011	DHS	LB2011MIS	361	854	779
Liberia	2013	DHS	LB2013DHS	435	1281	1005
Liberia	2016	DHS	LB2016MIS	509	716	672
Liberia	2019	DHS	LB2019DHS	537	834	642
Liberia	2022	DHS	LB2022MIS	573	504	449
Madagascar	2008	DHS	MD2008DHS	296	172	182

Madagascar	2011	DHS	MD2011MIS	396	102	98
Madagascar	2012	MICS	2012MICS	234	135	140
Madagascar	2013	DHS	MD2013MIS	456	56	63
Madagascar	2016	DHS	MD2016MIS	505	64	74
Madagascar	2018	MICS	MDG2018MICS	303	269	241
Madagascar	2021	DHS	MD2021DHS	560	254	230
Malawi	2006	MICS	MWI2006MICS	155	2051	1972
Malawi	2010	DHS	MW2010DHS	333	2938	2801
Malawi	2012	DHS	MW2012MIS	432	215	243
Malawi	2013	MICS	MWI2013MICS	246	3165	2966
Malawi	2014	DHS	MW2014MIS	473	232	285
Malawi	2015	DHS	MW2015DHS	483	1847	1855
Malawi	2017	DHS	MW2017MIS	512	196	307
Malawi	2019	MICS	MWI2019MICS	343	1806	1990
Mali	2006	DHS	ML2006DHS	276	662	693
Mali	2009	MICS	MLI2009MICS	182	941	995
Mali	2012	DHS	ML2012DHS	405	208	204
Mali	2015	DHS	ML2015MIS	487	573	564
Mali	2015	MICS	MLI2015MICS	275	444	439
Mali	2018	DHS	ML2018DHS	517	251	276
Mali	2021	DHS	ML2021MIS	574	91	91
Mauritania	2007	MICS	MRT2007MICS	173	250	279
Mauritania	2011	MICS	MRT2011MICS	213	145	160
Mauritania	2015	MICS	MRT2015MICS	274	145	202
Mauritania	2020	DHS	MR2020DHS	553	338	299
Mozambique	2008	MICS	MOZ2008MICS	176	985	1027
Mozambique	2011	DHS	MZ2011DHS	362	389	463
Mozambique	2018	DHS	MZ2018MIS	527	396	475
Namibia	2013	DHS	NM2013DHS	363	93	113
Niger	2006	DHS	NI2006DHS	277	751	760
Niger	2012	DHS	NI2012DHS	407	351	360
Niger	2021	DHS	NI2021MIS	575	247	316
Nigeria	2007	MICS	NGA2007MICS	172	1067	1044
Nigeria	2008	DHS	NG2008DHS	302	1133	1164
Nigeria	2010	DHS	NG2010MIS	392	820	883
Nigeria	2011	MICS	NGA2011MICS	212	2027	2083
Nigeria	2013	DHS	NG2013DHS	438	1217	1202
Nigeria	2015	DHS	NG2015MIS	474	1046	1026
Nigeria	2016	MICS	NGA2016MICS	290	2429	2605
Nigeria	2018	DHS	NG2018DHS	528	1567	1529
Nigeria	2021	DHS	NG2021MIS	576	590	572

Rwanda	2010	DHS	RW2010DHS	364	143	150
Rwanda	2013	DHS	RW2013MIS	449	91	98
Rwanda	2015	DHS	RW2015DHS	468	156	168
Rwanda	2017	DHS	RW2017MIS	540	155	169
Rwanda	2019	DHS	RW2019DHS	554	122	124
Sao Tome and Principe	2008	DHS	ST2008DHS	318	12	14
Sao Tome and Principe	2014	MICS	STP2014MICS	258	12	9
Sao Tome and Principe	2019	MICS	STP2019MICS	323	13	11
Senegal	2006	DHS	SN2006MIS	293	346	321
Senegal	2008	DHS	SN2008MIS	338	422	333
Senegal	2010	DHS	SN2010DHS	365	195	220
Senegal	2012	DHS	SN2012DHS	423	66	67
Senegal	2014	DHS	SN2014DHS	457	31	46
Senegal	2015	DHS	SN2015DHS	489	38	31
Senegal	2015	MICS	2015MICS	280	18	23
Senegal	2016	DHS	SN2016DHS	524	39	37
Senegal	2017	DHS	SN2017DHS	534	149	114
Senegal	2018	DHS	SN2018DHS	580	23	23
Senegal	2019	DHS	SN2019DHS	581	15	10
Sierra Leone	2005	MICS	SLE2005MICS	136	932	939
Sierra Leone	2008	DHS	SL2008DHS	324	290	279
Sierra Leone	2010	MICS	SLE2010MICS	188	1864	1872
Sierra Leone	2013	DHS	SL2013DHS	450	1322	1269
Sierra Leone	2016	DHS	SL2016MIS	515	938	866
Sierra Leone	2017	MICS	SLE2017MICS	292	1197	1248
Sierra Leone	2019	DHS	SL2019DHS	545	861	831
Somalia	2006	MICS	SOM2006MICS	153	88	95
Somalia	2011	MICS	2011MICS	207	33	32
Somalia	2011	MICS	2011MICS	208	90	89
South Sudan, Republic of	2010	MICS	SSD2010MICS	187	1228	1252
Sudan	2010	MICS	SDN2010MICS	185	735	688
Tanzania	2007	DHS	TZ2007AIS	304	615	745
Tanzania	2010	DHS	TZ2010DHS	345	864	1090
Tanzania	2012	DHS	TZ2012AIS	393	831	882
Tanzania	2015	DHS	TZ2015DHS	485	760	874
Tanzania	2017	DHS	TZ2017MIS	529	531	518
Tanzania	2022	DHS	TZ2022DHS	578	280	344
Togo	2006	MICS	TGO2006MICS	149	335	337
Togo	2010	MICS	TGO2010MICS	183	567	522
Togo	2013	DHS	TG2013DHS	328	306	262
Togo	2017	DHS	TG2017MIS	497	203	185

Togo	2017	MICS	TGO2017MICS	291	403	395
Uganda	2006	DHS	UG2006DHS	266	1884	1899
Uganda	2009	DHS	UG2009MIS	332	953	910
Uganda	2011	DHS	UG2011DHS	399	1893	2012
Uganda	2014	DHS	UG2014MIS	484	1061	1053
Uganda	2016	DHS	UG2016DHS	504	3730	3559
Uganda	2018	DHS	UG2018MIS	549	1221	1054
Zambia	2007	DHS	ZM2007DHS	278	447	432
Zambia	2013	DHS	ZM2013DHS	406	1195	1017
Zambia	2018	DHS	ZM2018DHS	542	544	471
Zimbabwe	2005	DHS	ZW2005DHS	260	24	21
Zimbabwe	2009	MICS	ZWE2009MICS	178	139	125
Zimbabwe	2010	DHS	ZW2010DHS	367	24	25
Zimbabwe	2014	MICS	ZWE2014MICS	253	87	79
Zimbabwe	2015	DHS	ZW2015DHS	475	12	10
Zimbabwe	2019	MICS	ZWE2019MICS	317	21	25

2.3.2 Surveys excluded from the final models

Six surveys were excluded from the final analysis and modelling. These comprised four MICS and two DHS, which were excluded for being small samples or for having estimates inconsistent with other survey results conducted within the same country. These surveys included one for Djibouti in 2006, one for Rwanda in 2008, another for Senegal in 2020 and two in Eswatini for 2010 and 2014, all of which had sample sizes falling below the threshold of 10 and insufficient for inclusion. Furthermore, a fourth MICS survey conducted in Mozambique in 2008 was excluded as an outlier due to an inconsistent estimate derived from the raw data compared to the value reported in the survey and further compared to other surveys conducted later within the same country (Table S2).

Table S2: Surveys excluded from the study

Country	Year	Program/ Source	Survey Name	Survey ID	Weighted No of Children	Reason for Exclusion
Djibouti	2006	MICS	DJI2006MICS	161	7	Small Sample
Eswatini	2010	MICS	SWZ2010MICS	196	3	Small Sample
Eswatini	2014	MICS	SWZ2014MICS	266	3	Small Sample
Rwanda	2008	DHS	RW2008DHS	323	3	Small Sample
Mozambique	2008	MICS	MOZ2008MICS	176	1027	Implausibility
Senegal	2020	DHS	SN2020MIS	587	1	Small Sample

Endemic countries with no survey data during the study period 2006-2022 (i.e., Botswana, Cape Verde, Eritrea, Ethiopia, Equatorial Guinea, and South Africa) had their fraction of treatment delay estimated using the modelling framework described in the main methods. Figure S2 summarizes the spatial distribution of countries with surveys explored in this analysis, including the number of eligible surveys included in the analysis.

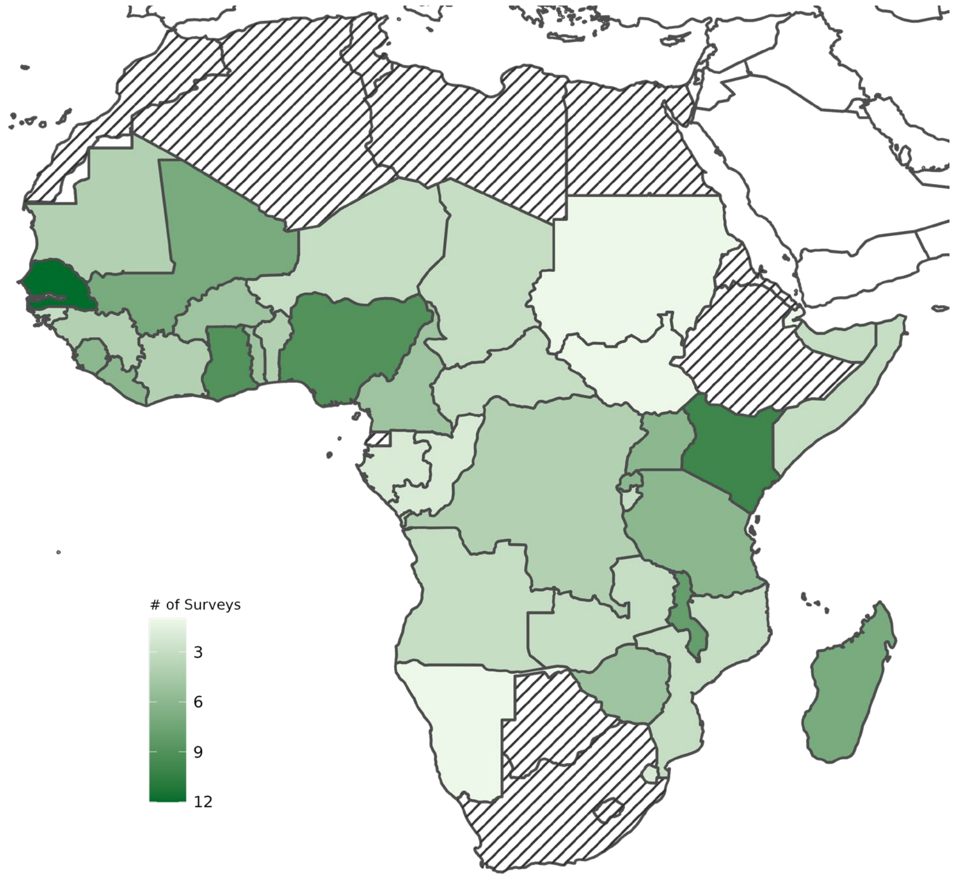

Figure S2: Spatial distribution and numbers of surveys modelled.

Countries without any surveys for the period under study are represented by hatches, while those with data are represented in shades of green with deeper green representing a bigger number of surveys available and lighter green for a smaller number of surveys available.

2.4 Data extraction process

The extraction from the master DHIS/MIS and MICS databases was done through the Malaria Survey Extraction tool, which was developed within the MAP Data Engineering team. All data and information from demographic surveys were consolidated at the child, caretaker/women, household, and country levels. For each malaria-endemic country survey

conducted on the African continent between 2006 and 2022, which collected data on the use of antimalarials, we extracted information based on responses on how long after the fever started, the child first took the antimalarial. An example of the respective question from the DHS Phase 7 (2013-2017) survey template would read as: How long after the fever started did (*child name*) first take the (*antimalaria named in a previous question)*? Responses to this question and its approved variations are usually numeric. Only children who reported febrile symptoms, received antimalarial treatment, and reported the time between the symptom onset and treatment were included. Figure S3 shows a summarized workflow of the inclusion and exclusion criteria for data extraction and curation.

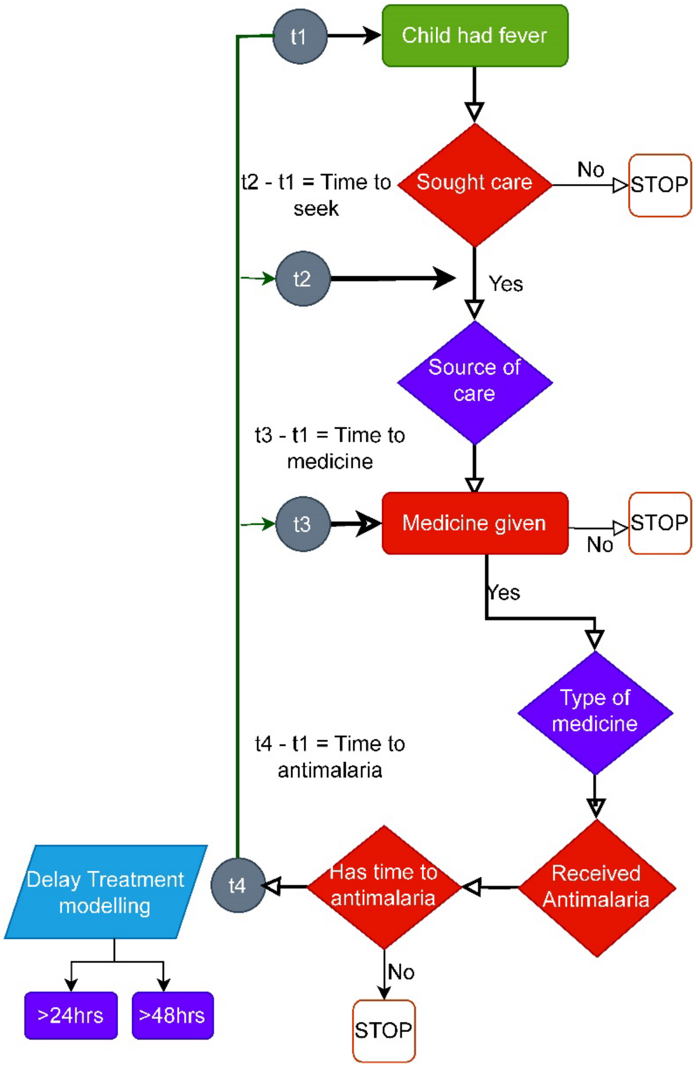

Figure S3: Schema of the inclusion and exclusion criteria for the surveys

The figure shows different stages of data extraction inclusion and exclusion criteria, where t represents specific significant time points in the care cascade and period between fever onset and receiving of antimalarial medication.*

2.4.1 Countries and WHO Africa subregions

The study also highlights both countries and World Health Organization (WHO) subregions of all endemic countries within Africa (Figure S4). The WHO categorization is done for modelling, analysis and often administration. The five WHO subregions include the WHO African Region (AFRO), comprised of central (AFRO-C), eastern (AFRO-E), southern (AFRO-S) and western (AFRO-W) African subregions, and the countries geographically within the African continent but administratively under the WHO Eastern Mediterranean Region (EMRO).

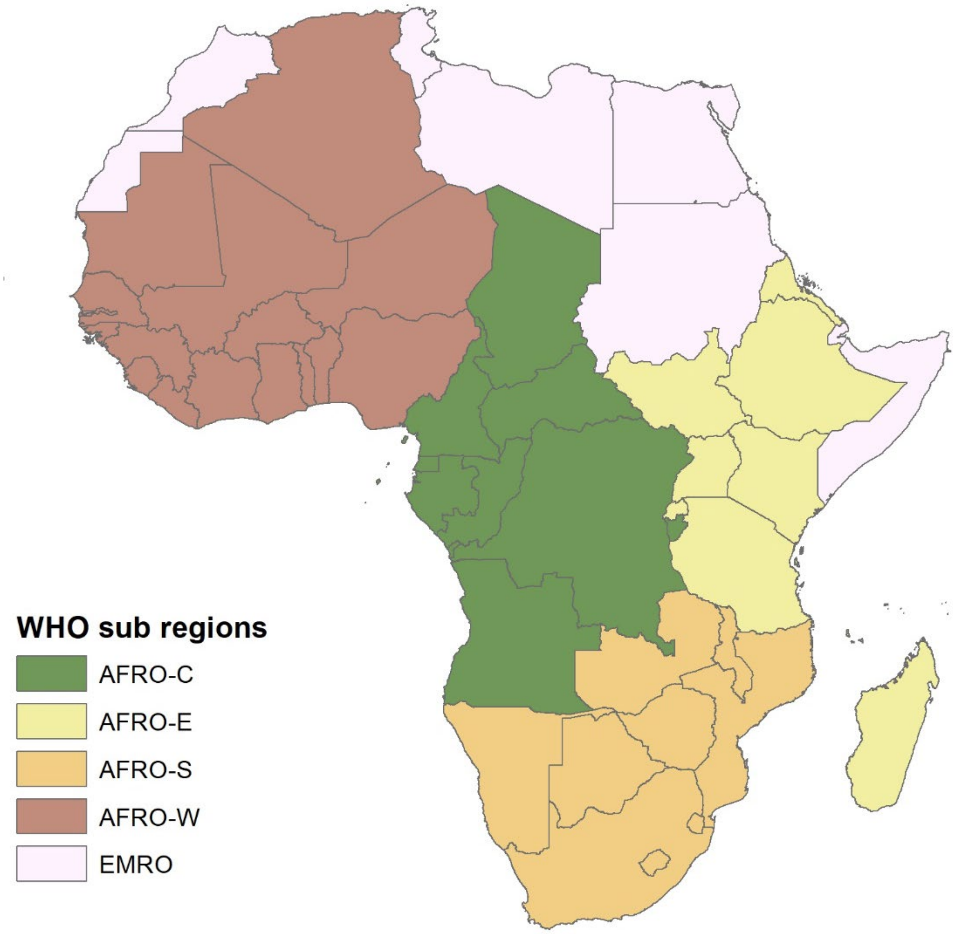

Figure S4*:* Country Subregional groupings used in the model

The figure shows regional groupings of countries and serves as both epidemiological and administrative groups recognised by the WHO.

2.5 Covariates

We performed a simple multivariate analysis using a generalised linear mixed model (GLM) as part of the exploratory data analysis. The analysis, based on pooled data, identified residence, wealth, mother's age, and education as key predictors of treatment delay. Urban residence, higher wealth, and education were associated with reduced treatment delays, while older age, multiple children under 5, and living in West and Central Africa were linked to increased delays. The child's sex and prior treatment received did not show significant effects and there was notable regional variation across countries. We used the results to initially determine the plausible covariates available for the final modelling using the Health Metrics and Evaluation (IHME), and the Malaria Atlas Project (MAP) datasets.

The analysis considered many variables and factors assumed to influence the access and delivery of effective malaria services from both the health systems as well as the patient's perspective. The initial set of variables that were considered in this modelling process was extracted from the IHME, and MAP covariate databases and supplemented by the World Health Organization (WHO).

IHME collates and produces country- and annual-level variables, including health system access and metrics that characterise socioeconomic status. MAP compiles, maintains, and generates gridded global and national-level covariate estimates of treatment-seeking rates and other interventions, while antimalarial data was compiled from WHO World Malaria Reports. A list of 30, mostly socioeconomic covariates, was collated, and a simple covariate selection process using generalised mixed models was explored. The covariate selection allowed for the selection of an enhanced set to be chosen before performing the final modelling step, allowing the model to estimate the fraction of delay more accurately. All covariates were nation-level covariates providing values for each country-year space-time matrix. Final model covariates were selected after exploring simple covariate selection generalised linear mixed models. Table S3 has detailed information on each of the covariates explored during the modelling process, showing if that covariate was used in the final model and providing traceable references.

Table S3: Summary of Covariates explored for modelling

Covariate	Description	Source	In final model	Reference
Antenatal Care (4 visits) Coverage (proportion)	Proportion of pregnant women receiving 4 or more antenatal care visits, including 1 or more from a skilled provider	GBD	NO	GBD covariate: "Proportion of pregnant woman receiving 4 or more antenatal care visits including 1 or more from a skilled provider"

DTP3 Coverage (proportion)	Fraction of children born in a given country-year who have received 3 doses of DTP3	GBD	NO	GBD Covariate: "Fraction of children born in a given country-year who have received 3 doses of DTP3"
Education age-standardized	Age-standardized level of educational attainment	GBD	NO	GBD Covariate: "Age-standardized level of educational attainment"
Education (years per capita)	Mean level of educational attainment	GBD	NO	GBD Covariate: "Mean level of educational attainment"
GDP per capita base 2010	GDP per capita base 2010 international dollars	GBD	NO	GBD Covariate: "GDP per capita base 2010 international dollars"
Hospital Beds (per 1000)	Hospital beds per 1000 people	GBD	NO	GBD Covariate: "Hospital beds per 1000 people"
In-facility delivery (proportion)	Percent of women giving birth in a health facility	GBD	NO	GBD Covariate: "Percent of women giving birth in a health facility"
Health Industry Workers	The proportion of the employed population ages 15-69 working in health and social work (according to ISIC classifications)	GBD	NO	GBD Covariate: "The proportion of the employed population ages 15-69 working in health and social work (according to ISIC classifications)"
Is The Antimalaria Chloroquine	Is Chloroquine first first-line malaria treatment	WHO	NO	WHO Covariate: "Is Chloroquine first-line malaria treatment"
Is The Antimalaria Other	Is Other antimalarials first-line malaria treatment	WHO	NO	WHO Covariate: "Is Other antimalarials first-line malaria treatment"
Is The Antimalaria Sulfadoxine/Pyrimethamine (Sp)	Is SP antimalarial first-line malaria treatment	WHO	NO	WHO Covariate: "Is SP antimalarial first-line malaria treatment"
LDI (I$ per capita)	Lag distributed income per capita (I$): gross domestic product per capita that has been smoothed over the preceding 10 years	GBD	NO	GBD Covariate: "Lag distributed income per capita (I$): gross domestic product per capita that has been smoothed over the preceding 10 years"
Malaria incidence (MAP)	P. falciparum incidence 1980-2016 national and subnational	MAP	NO	Bhatt, S. et al. The effect of malaria control on Plasmodium falciparum in Africa between 2000 and 2015. Nature 526, 207–211 (2015).
Measles Vaccine Coverage (proportion)	Percentage of the population with measles vaccination	GBD	NO	GBD Covariate: "Percentage of population with measles vaccination"
Measles Vaccine Coverage 2 doses (proportion)	Percentage of population with 2 doses of measles vaccination	GBD	NO	GBD Covariate: "Percentage of population with 2 doses of measles vaccination"
All age underweight	All age prevalence of underweight from adult_underweight covariate	GBD	NO	GBD Covariate: "All age prevalence of underweight from adult_underweight covariate"

Universal health coverage	Coverage of universal health coverage tracer interventions for prevention and treatment services, percent; created for GBD 2015 SDGs paper.	GBD	NO	GBD Covariate: "Coverage of universal health coverage tracer interventions for prevention and treatment services, percent; created for GBD 2015 SDGs paper."
Nighttime lights	Nighttime Lights Imagery for Measuring Urbanization Trends in Africa	NOAA DMSP	NO	Savory et al. Intercalibration and Gaussian Process Modelling of Nighttime Lights Imagery for Measuring Urbanization Trends in Africa 2000–2013. Remote Sens. 9, (2017).
Antenatal Care (1 visit) Coverage (proportion)	Proportion of pregnant women receiving any antenatal care from a skilled provider	GBD	YES	GBD Covariate: "Proportion of pregnant women receiving any antenatal care from a skilled provider"
Education (years per capita) aggregated by age (15+) and sex	Education (years per capita) aggregated by age (15+) and sex	GBD	YES	GBD Covariate: "Education (years per capita) aggregated by age (15+) and sex"
Healthcare access and quality index	Healthcare access and quality index	GBD	YES	GBD Covariate: "Healthcare access and quality index"
Maternal care and immunization	A measure of health system access estimated using a principal component analysis of antenatal clinics, DTP3 immunization, measles immunization, in-facility delivery, and skilled birth attendance. Max value for each year capped to min OECD value	GBD	YES	GBD Covariate: "A measure of health system access estimated using a principal component analysis of antenatal clinics, DTP3 immunization, measles immunization, in-facility delivery, and skilled birth attendance. Maximum value for each year capped to min OECD value."
Health expenditure (per capita)	The variable is health expenditure per capita taken from FGH April 2019, in 2018 USD	GBD	YES	GBD Covariate: "The variable is health expenditure per capita taken from FGH April 2019, in 2018 USD"
Is Artemisinin-Based Combination Therapy	Is ACT first-line malaria treatment	WHO	YES	WHO Covariate: "Is ACT first-line malaria treatment"
Log total health expenditure per capita	Log-transformed national-level estimates for total health expenditure per capita	GBD	YES	GBD Covariate: "Log-transformed national-level estimates for total health expenditure per capita"
Fraction of OOP Health Expenditure	Fraction of out-of-pocket health expenditure out of total health expenditure from FGH April 2019	GBD	YES	GBD Covariate: "Fraction of out-of-pocket health expenditure out of total health expenditure, from FGH April 2019"
Urbanicity	Urbanicity	European Commission/GHS	YES	Pesaresi, M. et al. Operating procedure for the production of the Global Human Settlement Layer from Landsat data of the epochs 1975, 1990, 2000, and 2014.

				(Publications Office of the European Union, 2016).
Skilled Birth Attendance (proportion)	Percent of women giving birth with a skilled birth attendant (mainly nurses, doctors, midwives)	GBD	YES	GBD Covariate: "Percent of women giving birth with a skilled birth attendant (mainly nurses, doctors, midwives)"
Treatment Seeking proportion	National-year treatment seeking proportion	MAP	YES	Nguyen M, et al. (2023) Trends in treatment-seeking for fever in children under five years old in 151 countries from 1990 to 2020. PLOS Glob Public Health 3(8): e0002134. doi.org/10.1371/journal.pgph.0002134

2.6 Distribution of fraction of treatment delay in reported estimates from pooled surveys

A pooled distribution of the fraction of treatment delay has also been reported, shown in Figure S5, where the fraction of treatment delay across the full spectrum of the data and the distribution of the three categories of days to receiving treatment, particularly <24 hours (prompt), moderate delay, and severe delay.

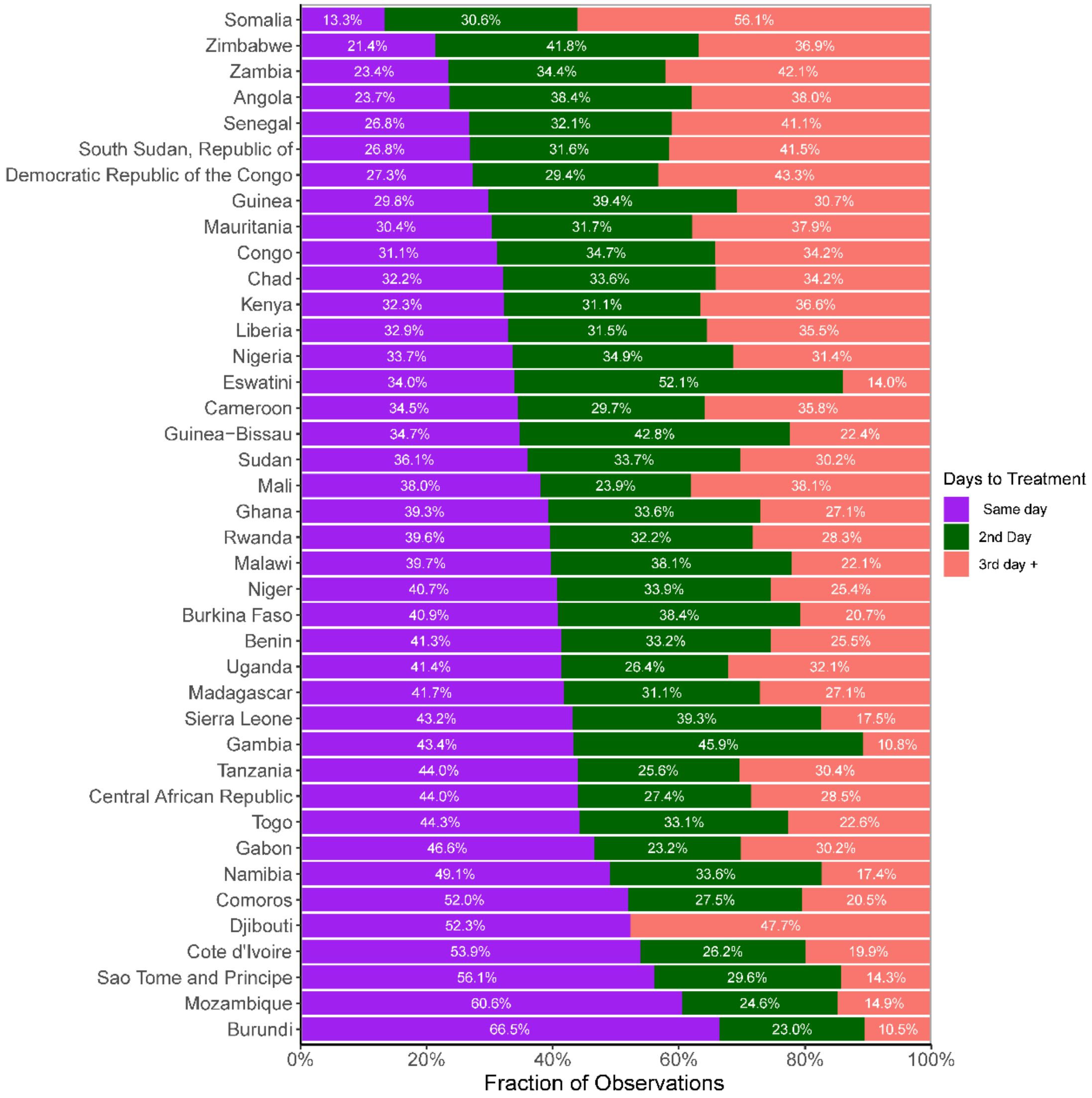

Figure S5*: Crude estimates of delay among treated cases based on pooled survey data*

Distribution of pooled raw fractions of treatments for malaria by treatment day of treatment, by country.

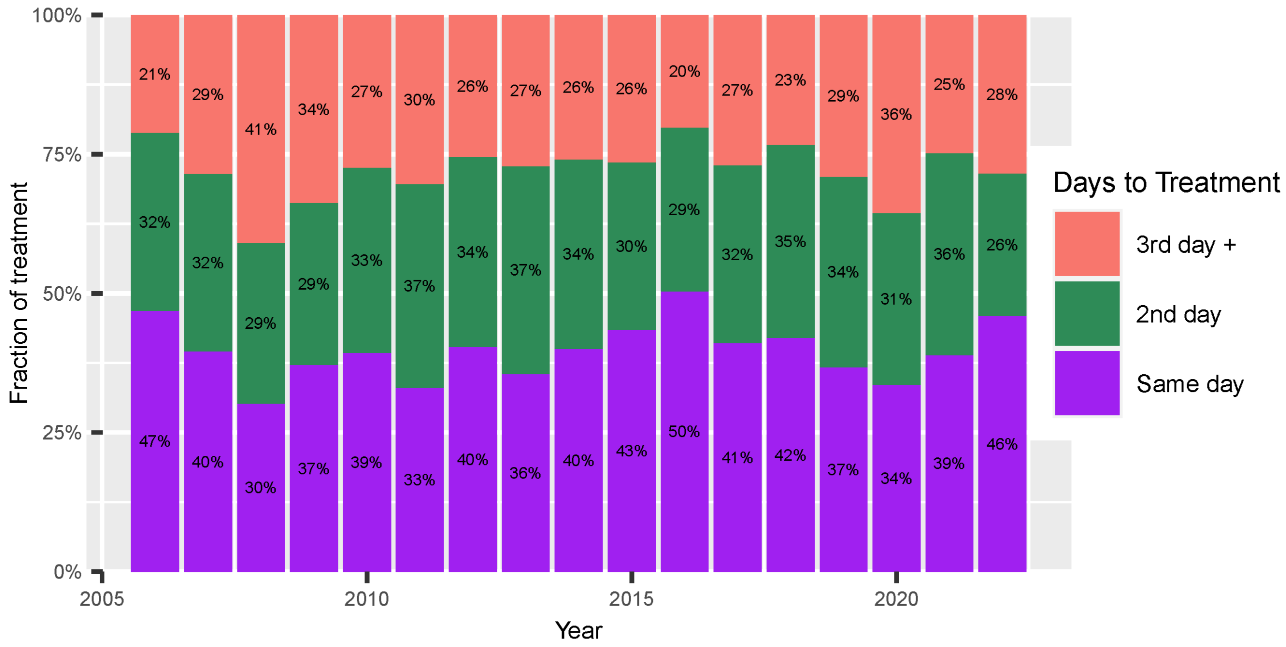

Figure S6*:* Pooled survey estimates of annual continent-wide delayed malaria treatment
The area plot of the continent-wide pooled distribution of raw data and trends of fractions of treatment delay between 2006 and 2022. Terms of delay are represented by separate colours.

2.7 Model fitting, scaling and control parameters

We used the Empirical Bayesian (eb) option for the intercept strategy in the INLA control parameter. To ensure the convergence and stability of the model, we scaled the fraction of treatment delay estimates appropriately. Additionally, we specified various control parameters, including configuration settings, computation of the Watanabe-Akaike information criterion (WAIC), Deviance Information Criterion (DIC) Conditional Predictive Ordinates (CPO), Probability Integral Transform (PIT) and the Marginal Likelihood (Mlik). A summary of model performance comparison is reported in Table S4.

Table S4: Summary of model performance metrics

Model	Name	WAIC	CPO	PIT	DIC	MLIK
Model 1	Binomial with log odds via Z & AR1	118.84	103.37	82.53	122	-171.68
Model 2	Binomial with AR1	1378.95	2.31	85.89	1413.7	-933.6
Model 3	Beta-binomial with Link & IID	1683.54	2.08	84.77	1682.94	-895.57
Model 4	Beta-binomial with Link & AR1	1620.36	2.71	82.42	1627.47	-901.02

Based on model performance comparison results presented in SI Table S4, the binomial model implemented with log odds via Z and AR1 (Model 1) consistently outperformed all other models across multiple performance metrics. Specifically, Model 1 had the lowest WAIC (118.84) and DIC (122), the highest CPO (103.37), and the least negative MLIK (-171.68), indicating superior model fit and predictive performance. In contrast, the direct

binomial model with AR1 (Model 2) and the beta-binomial models (Models 3 and 4) exhibited substantially higher WAIC, DIC, and more negative MLIK values, suggesting poorer performance. The PIT values were comparable across models, with Model 1 showing a slight advantage. These results justify the selection of Model 1 as the optimal model for this analysis.

2.8 Validation and accuracy

A summary of model validation and overall consistent fit is provided in Figures S7a and S7b. The point difference between survey estimates and modelled estimates of > 10% was inspected to ensure we understood why the given country-year estimates differed significantly. Most of these discrepancies occurred in countries and surveys with little consistency between survey series. For example, surveys from Zimbabwe (2014 and 2015) and Senegal (2016 and 2018) show huge fluctuations in the fraction of delayed treatment over unrealistically short timespans.

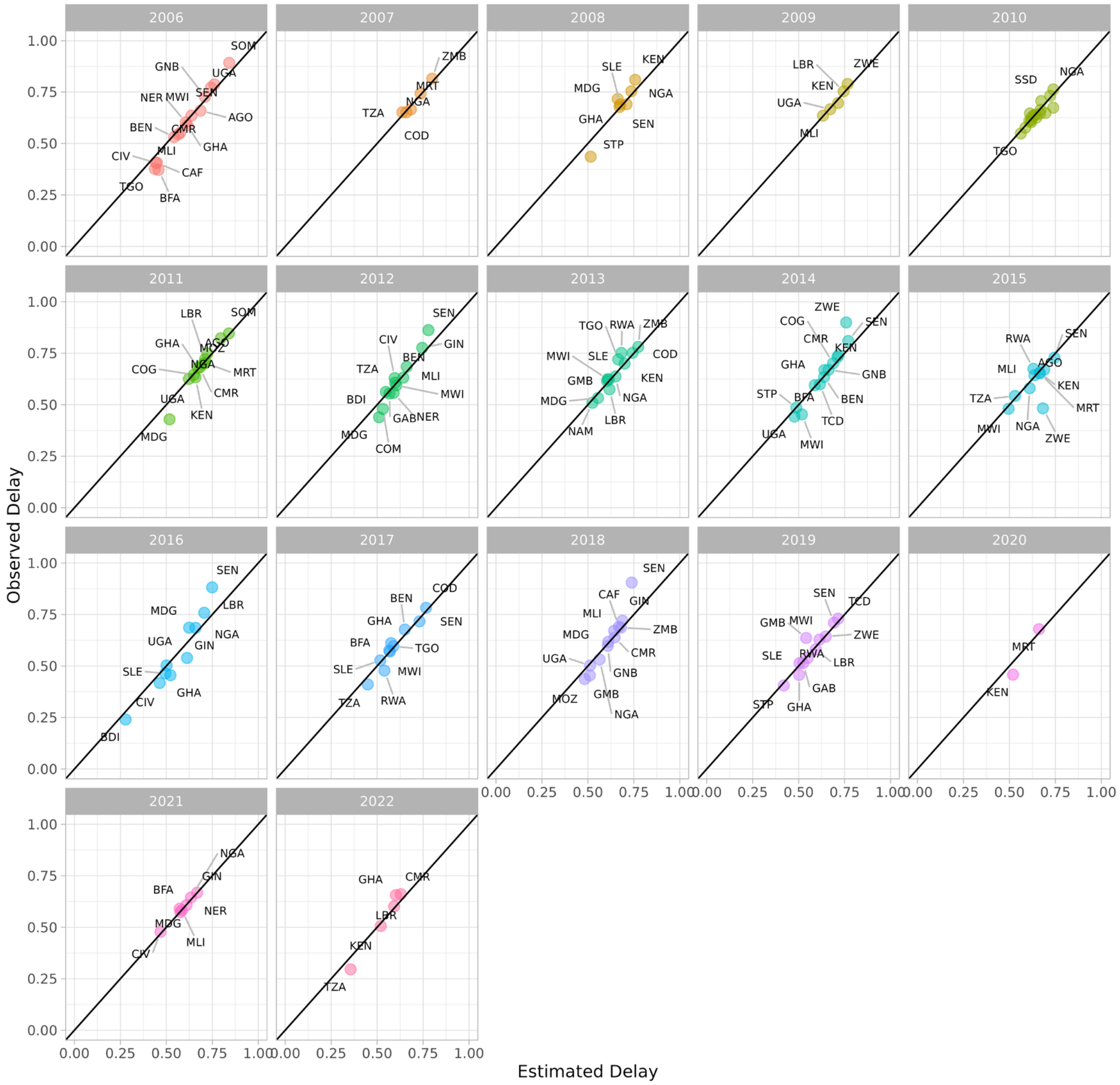

Figure S7a: Modelled vs observed survey estimates for fraction of treatment delay >24 hours

Model performance comparison by year.

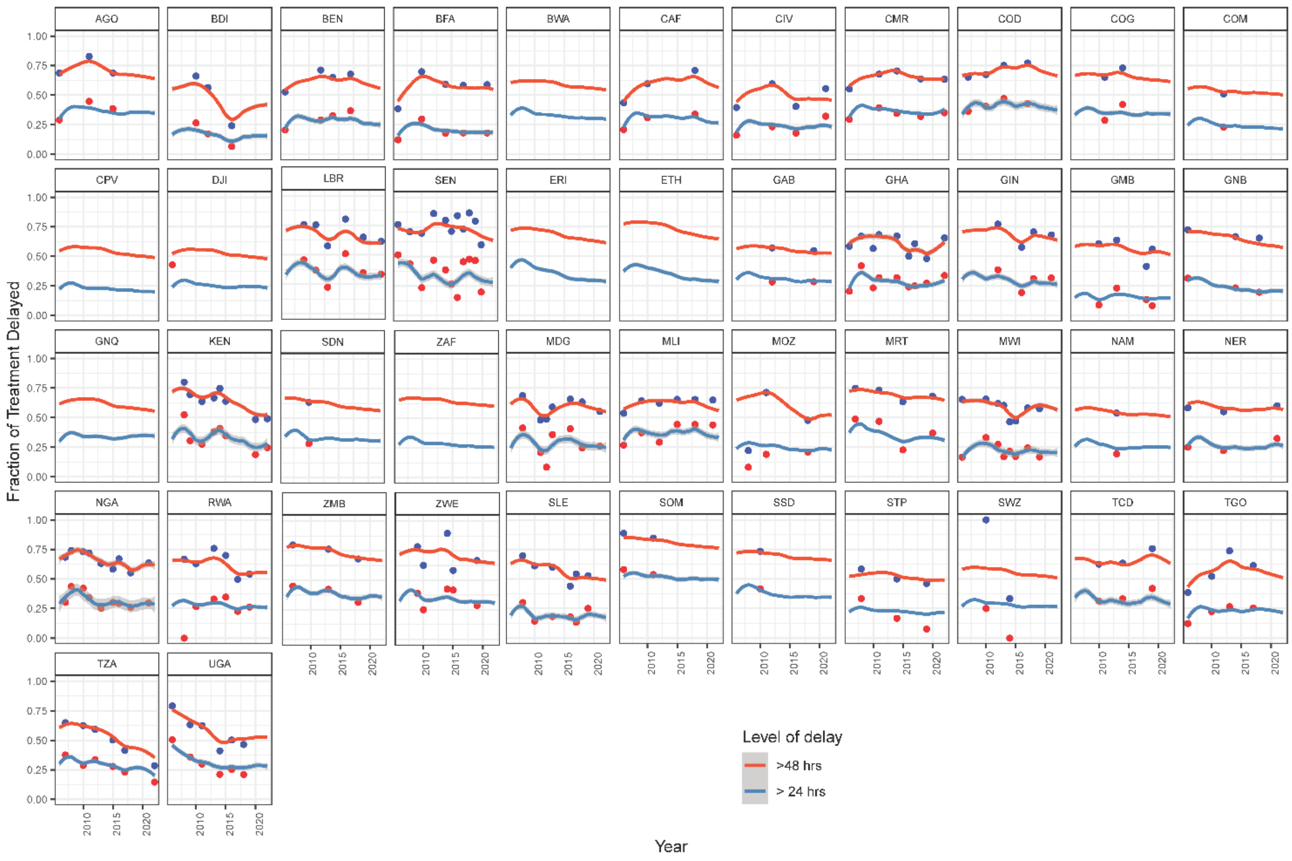

Figure S7b: Overall model fit comparing observed vs modelled estimates across delay levels and country-years. All survey estimates (including those excluded from the models) have been added to compare with modelled estimates. You can thus still compare the modelled estimates for Senegal 2020, Rwanda, Eswatini 2010 and 2014, and Mozambique 2008 against what their skewed survey estimates from samples less than 10 gave.

3. Supplementary Results

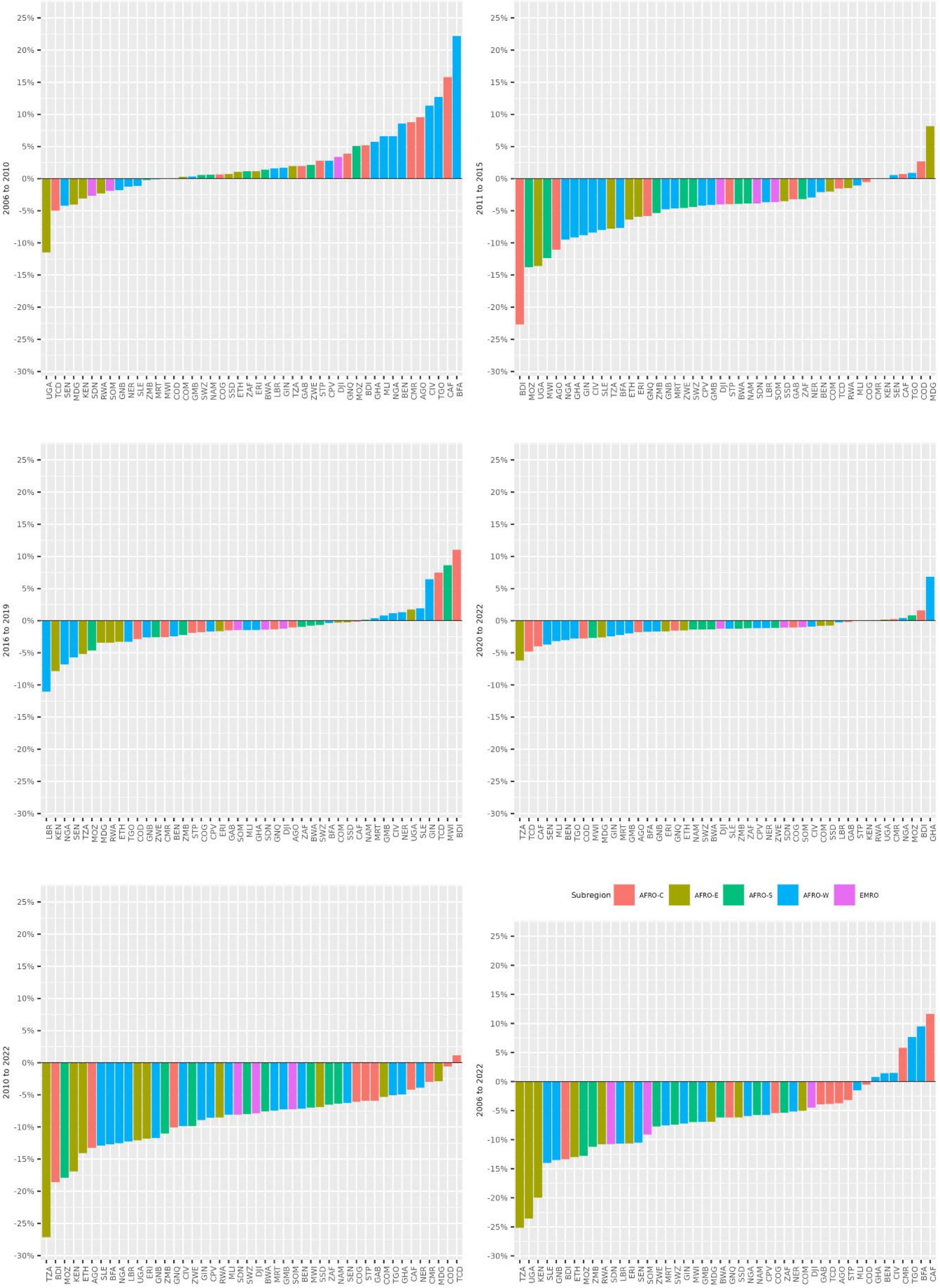

Figure S8: Country progress in delay between 2006-2010, 2011-2015, 2016-2020, 2021 – 2022, and overall and 2006-2022
The different colours represent subregions to which a country belongs, and the bars extending downwards represent the % decline in delayed treatment, while those extending above 0 represent an increase in delay between two time points.

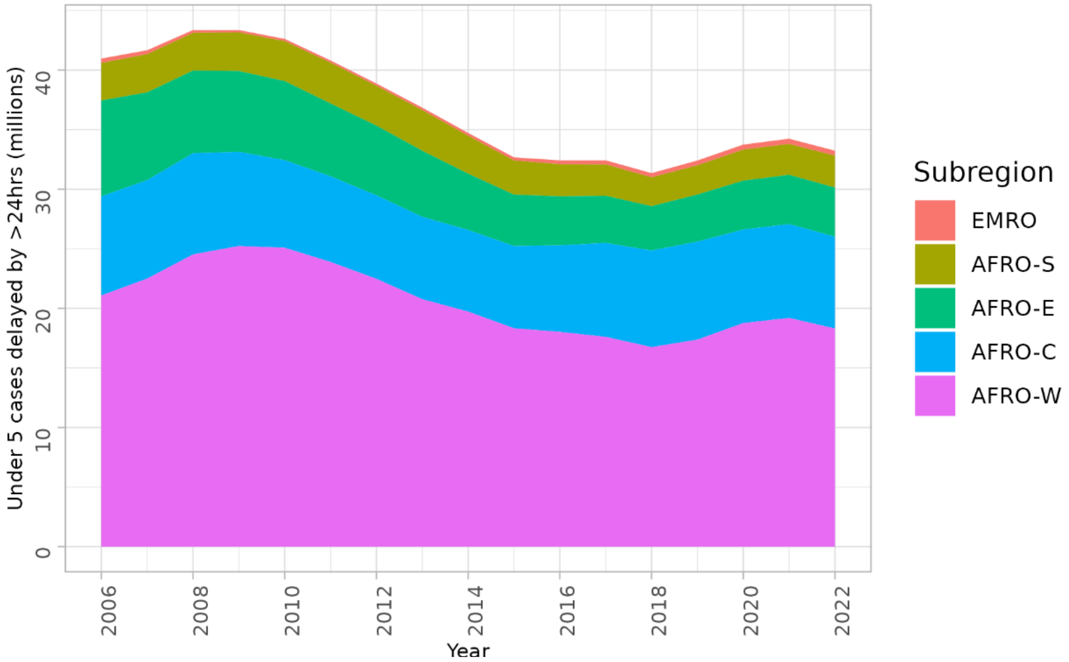

Figure S9*:* Subregional trends of treated cases delayed by >24 hours

The different colours represent subregions to which a country belongs, while the cumulative layers represent the share of cases by each region.

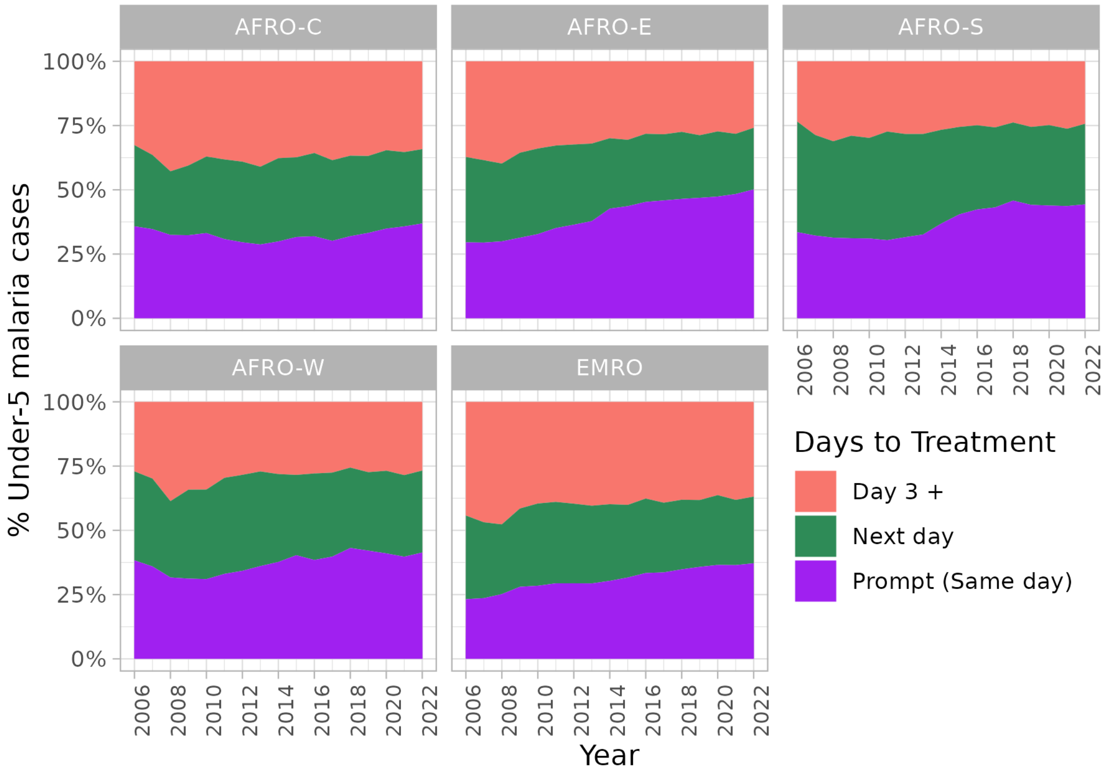

Figure S10: Trends of subregional fraction based on days to treatment

Purple represents same-day treatment after the onset of symptoms; Green represents treatment obtained the following day [moderate delay] after the onset of symptoms and orange represents severe delay treatment

(a) Subregion contribution to delayed treatment among U5 children in 2022

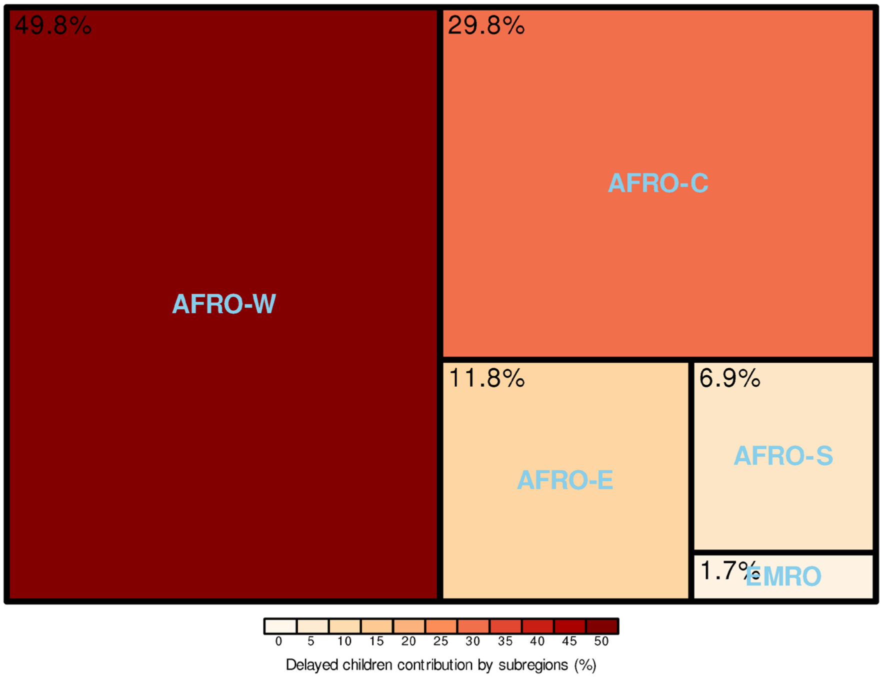

(b) Country contribution to fraction (%) of all treated children delayed in 2022

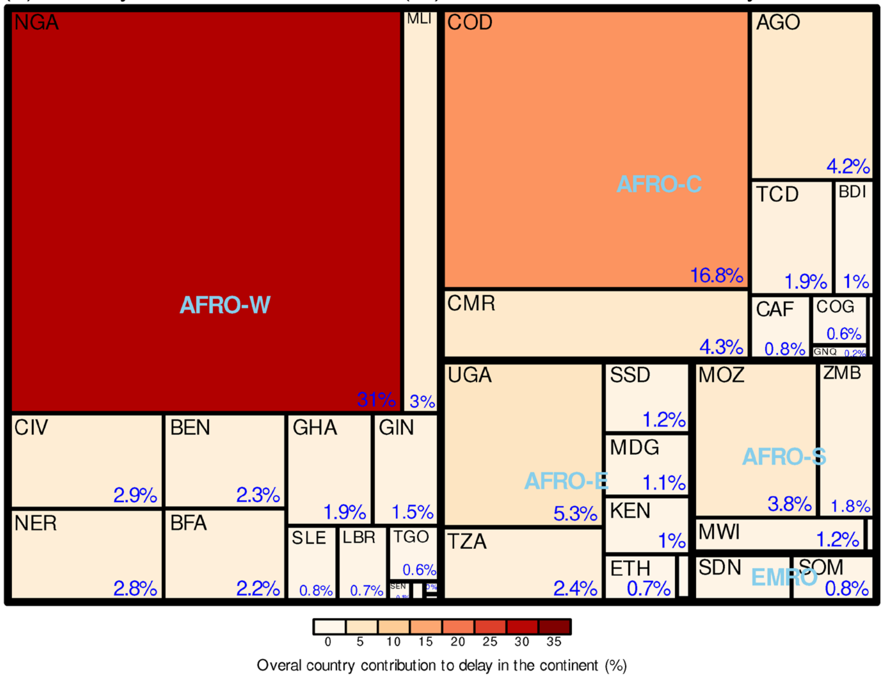

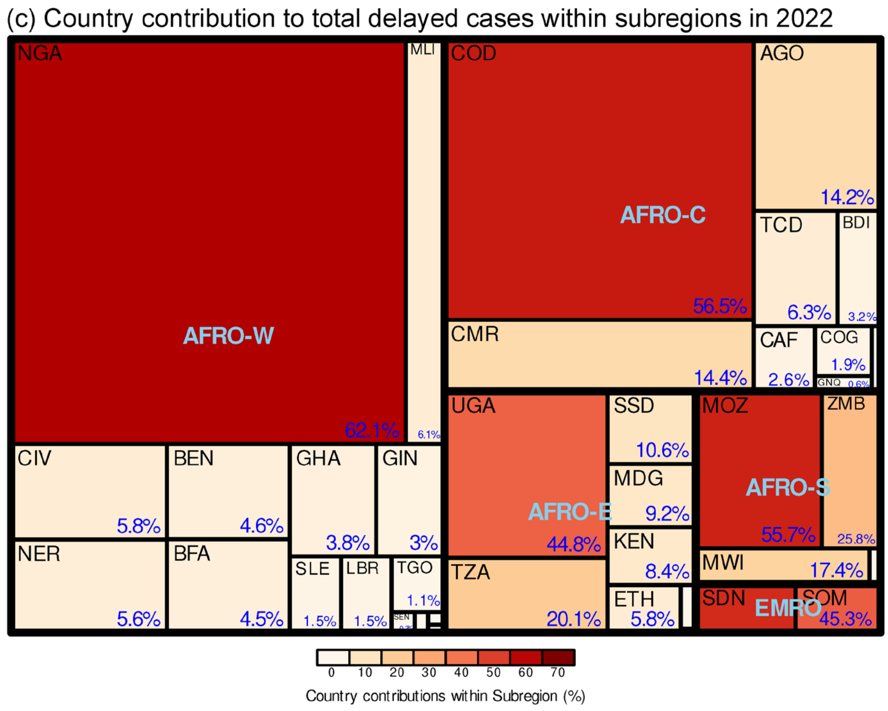

Figure S11(A)-(C): Contribution (%) of countries and subregions to delayed cases.
In B and C, thicker lines = Subregion (all countries belonging to the same subregion), Label positions: Country code = *top left corner*; % contribution = *bottom right corner*; Subregions labels are centred.

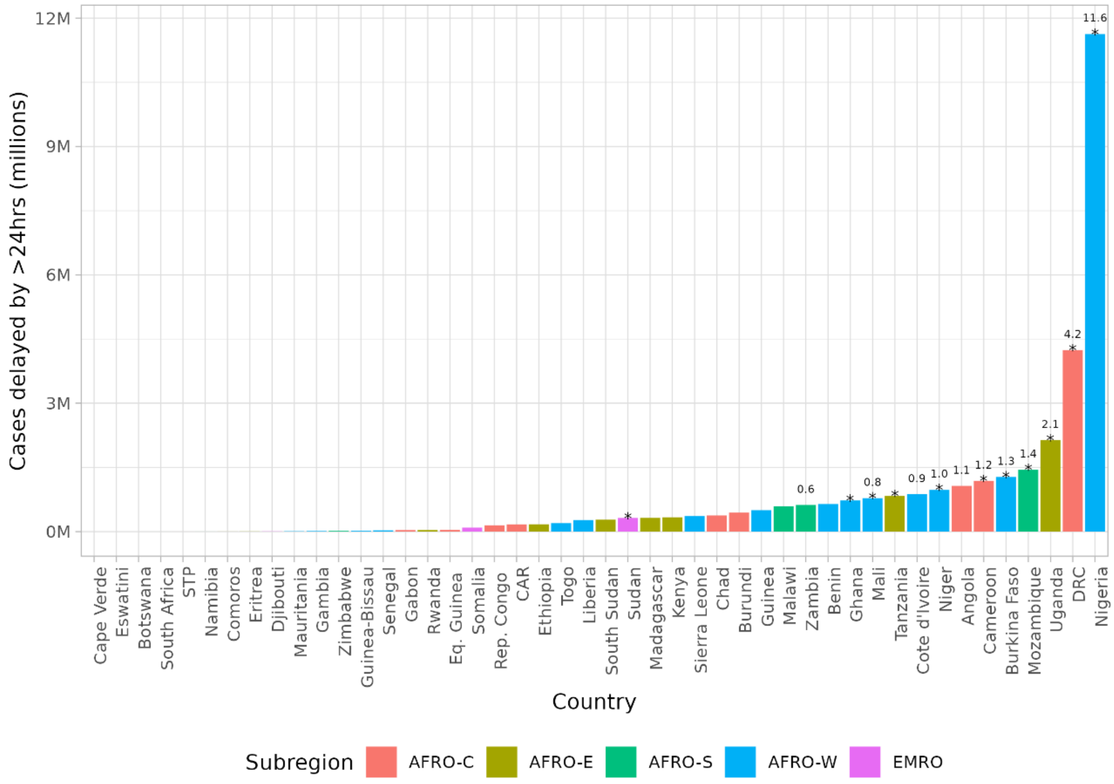

Figure S12: Countries ordered by estimated delayed cases by >24 hours
The different colours represent subregions to which a country belongs, and the "" denotes an HBHI country.*

Figure S12 shows cases of under-5 treated delayed by >24 hours. The top 10 countries with the most number of delayed febrile cases among children are dominated by 80% (8/10) of HBHI countries in Africa. The figure estimates 57 million treated child febrile cases delayed in receiving antimalarial treatment.

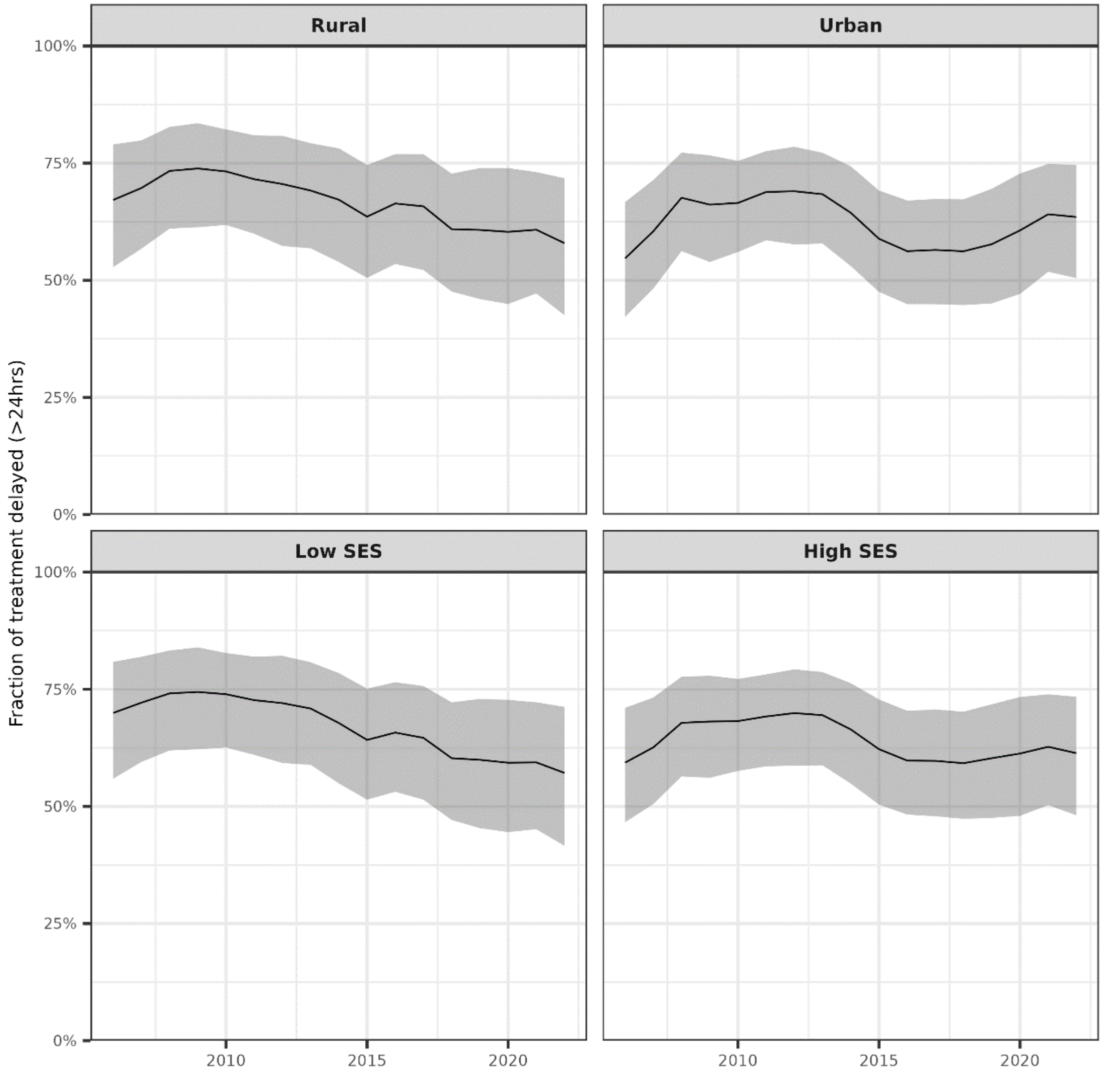

Figure S13: Continent-wide trends of rural vs urban and poor vs wealthier households
The Figure shows trends differences between socioeconomic groups; and rural-urban households, presented within upper UI and Lower UI.

3.1 Periodic Temporal Trends between 2006-2010, 2011-2015, 2016-2022

Overall, 85% (39/46) of the countries made progress between 2006 and 2022, ranging between 1% and 27% declines, while 15% (7/46) of them experienced more treatment delay

in 2022 compared to 2006. When broken into four time periods: 2006-2010, 2011-2015, 2016-2020, and 2021-2022, most malaria treatment delay progress happened in the period from 2011-2015, while 2016-2020 and 2020-2022 had mostly marginal declines, respectively. Figure S14 shows the overall pooled progress made at the continental level in the different time periods of the study. This is complemented by Figure S8, which shows how each country performed during the periodic classes, including which countries had declines or increases in delay and the % progress relative to other countries.

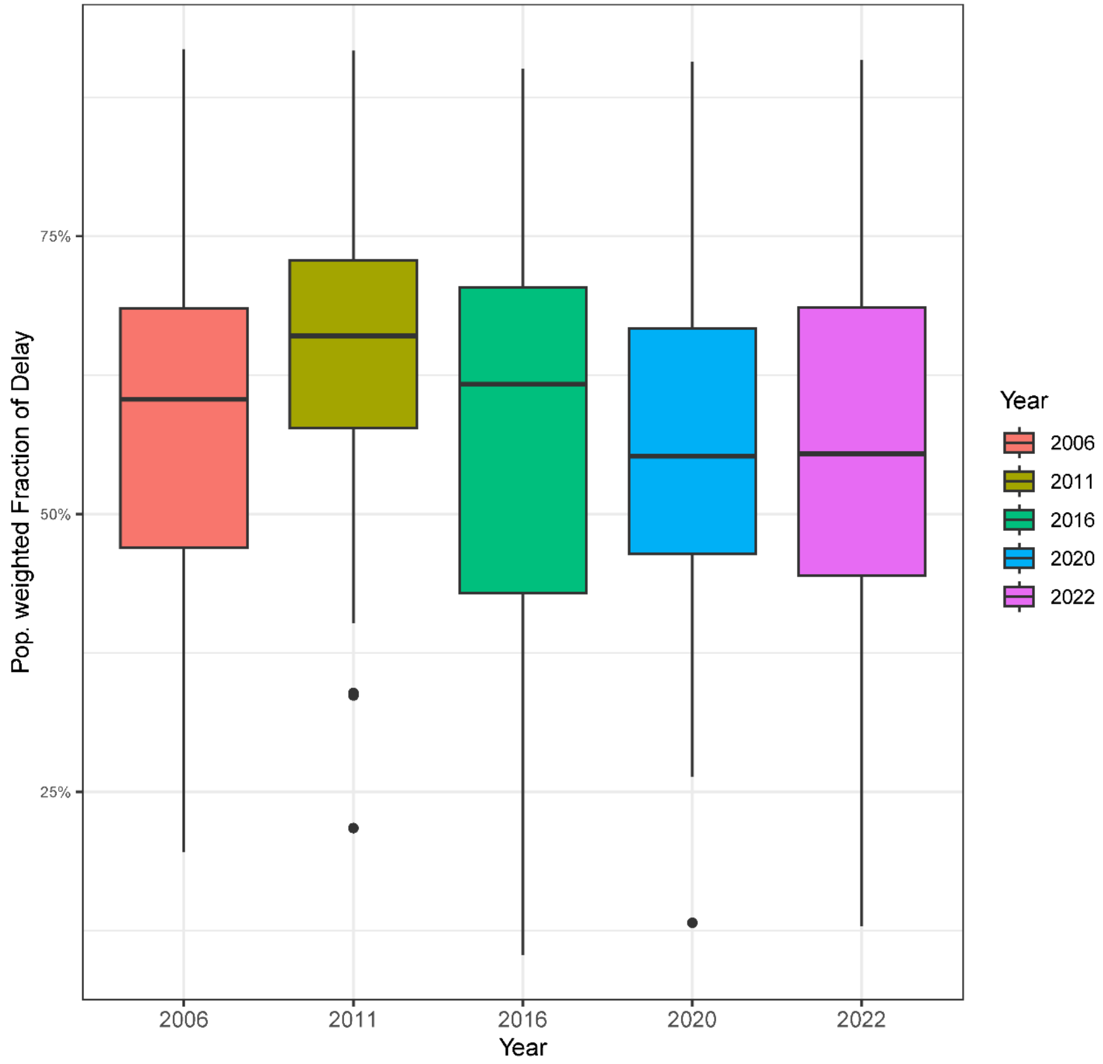

Figure S14: Periodic progress made in treatment delay

Box plots population-weighted distribution of delay estimates with each colour representing a unique 5-year time points before 2020 and 3 years for the last category.

Table S5: Fraction of severe delay estimates

Country	2006	2007	2008	2009	2010	2011	2012	2013	2014	2015	2016	2017	2018	2019	2020	2021	2022
Angola	29.96% (9.83 - 52.05)	32.48% (8.26 - 70.04)	34.58% (9.25 - 74.89)	37.06% (12.27 - 78.78)	38.35% (14.86 - 79.39)	40.13% (26.82 - 72.41)	37.21% (14.42 - 78.09)	36.04% (12.86 - 77.6)	35.11% (12.96 - 75.81)	35.53% (20.13 - 67.25)	34.43% (12.07 - 76.51)	34.81% (11.73 - 79.22)	36.17% (12.43 - 81.83)	36.33% (13.52 - 82.43)	35.87% (13.09 - 82.5)	35.29% (12.97 - 81.52)	34.94% (12.23 - 81.73)
Benin	21.72% (5.36 - 26.74)	26.94% (5.26 - 54.84)	28.68% (5.9 - 62.63)	28.87% (6.25 - 63.07)	29.58% (6.77 - 64.73)	28.47% (6.56 - 61.33)	26.95% (9.27 - 44.69)	29.41% (8.02 - 61.88)	32.62% (16.76 - 58.39)	29.66% (8.33 - 64.3)	29.22% (7.94 - 65.23)	33.23% (17.74 - 63.06)	28.13% (7.52 - 63.82)	25.98% (5.77 - 60.37)	24.68% (5.07 - 59.36)	24.32% (5.16 - 58.37)	23.57% (4.7 - 57.73)
Botswana	36.02% (7.59 - 80.97)	36.05% (7.66 - 82.12)	36.15% (7.08 - 84.03)	35.45% (7.04 - 83.25)	33.39% (6.27 - 79.08)	33.9% (6.82 - 81.11)	32.94% (6.71 - 79.23)	32.44% (6.62 - 78.87)	31.5% (6.3 - 77.54)	31.51% (6.18 - 78.48)	31.09% (6.09 - 78.79)	29.97% (5.58 - 76.6)	29.74% (5.42 - 76.93)	29.43% (5.66 - 77.53)	28.9% (5.65 - 76.76)	28.76% (5.61 - 76.24)	28.1% (5.27 - 75.96)
Burkina Faso	14.61% (2.22 - 16.92)	19.32% (2.81 - 38.49)	22.11% (3.66 - 49.52)	22.87% (4.59 - 51)	27.77% (12.3 - 50.09)	22.88% (4.53 - 52.11)	21.09% (3.68 - 47.87)	20.24% (3.57 - 45.07)	19.58% (5.72 - 31.54)	20.02% (3.65 - 45.59)	19.53% (3.51 - 44.87)	18.5% (5.15 - 31.13)	18.72% (3.26 - 43.65)	18.7% (3.1 - 47.29)	18.38% (3.05 - 46.17)	18.43% (3 - 46.98)	18.02% (2.98 - 46.99)
Burundi	17.75% (2.08 - 39.1)	18.1% (2.34 - 40.4)	18.87% (2.73 - 42.52)	18.99% (3.06 - 41.17)	22.5% (7.66 - 40.59)	18.71% (3.43 - 40.06)	17.41% (4.23 - 27.47)	16.13% (2.29 - 34.59)	14.43% (1.69 - 31.14)	12.28% (1.28 - 22.36)	8.27% (0.96 - 6.74)	12.39% (1.32 - 23.86)	13.78% (1.62 - 30.37)	14.55% (1.86 - 34.5)	14.64% (1.96 - 35.55)	14.77% (2.02 - 36.72)	14.62% (2 - 36.75)
Cameroon	31.3% (12.22 - 50.87)	34.94% (10.3 - 71.78)	36.5% (11.16 - 76.02)	37.02% (11.9 - 76.43)	36.76% (12.84 - 76.25)	38.99% (24.04 - 69)	37.22% (14.04 - 77.5)	36.33% (12.92 - 76.06)	36.22% (21.06 - 66.13)	35.22% (12.82 - 75.21)	34.32% (11.5 - 75.31)	34.03% (11.71 - 74.15)	33.37% (17.67 - 62.87)	32.46% (10.74 - 72.71)	31.58% (9.71 - 72.39)	31.42% (9.41 - 73.11)	30.86% (9.35 - 73.47)
Cape Verde	22.08% (1.69 - 56.85)	22.48% (1.7 - 58.17)	22.04% (1.66 - 59.72)	21.64% (1.63 - 59.86)	20.17% (1.47 - 53.36)	20.36% (1.45 - 55.41)	20.09% (1.5 - 55.47)	19.65% (1.3 - 55.78)	19.86% (1.27 - 57.95)	19.46% (1.19 - 57.97)	18.93% (1.13 - 57.99)	18.93% (1.21 - 58.52)	18.76% (1.2 - 57.8)	17.45% (1.13 - 51.97)	17.03% (1 - 52.64)	17.14% (1.14 - 54.36)	16.8% (0.97 - 54.82)
Central African Republic	22.24% (6.52 - 33.79)	28.25% (7.33 - 62.8)	30.98% (8.42 - 71.06)	31.24% (9.56 - 70.36)	32.3% (17.53 - 60)	31.16% (9.43 - 72.8)	30.87% (9.1 - 73.75)	31.39% (9.42 - 76.13)	30.64% (9.16 - 75.2)	29.91% (8.76 - 74.02)	30.47% (9.54 - 73.76)	29.99% (10.23 - 72)	32.85% (21.23 - 66.25)	27.47% (8.22 - 68.57)	25.79% (6.62 - 67.76)	25.15% (6.38 - 66.86)	24.33% (5.84 - 66.6)
Chad	37.83% (14.02 - 83.43)	37.54% (14.51 - 82.95)	36.98% (14.42 - 81.26)	33.94% (12.75 - 76.63)	28.88% (14.52 - 54.06)	30.81% (10.14 - 71.78)	31.09% (9.84 - 74.57)	30.94% (10.66 - 72.99)	28.2% (14.2 - 54.56)	29.5% (9.54 - 70.98)	30.09% (9.82 - 74.1)	31.18% (10.84 - 77.19)	33.14% (13.99 - 78.92)	37.62% (32.13 - 74.99)	30.96% (12.12 - 76.39)	29.46% (10.17 - 76.34)	28.54% (8.85 - 75.49)
Comoros	28.04% (5.3 - 69.63)	28.85% (5.89 - 70.75)	28.71% (6.13 - 71.7)	28.04% (6.23 - 69.53)	27.03% (6.16 - 66.88)	25.81% (5.89 - 61.51)	22.46% (6.8 - 42.5)	23.87% (5.46 - 58.47)	24.2% (5.22 - 62.74)	23.8% (4.77 - 63.68)	23.15% (4.63 - 61.98)	23.37% (4.8 - 64.17)	23.3% (4.82 - 62.96)	22.75% (4.61 - 62.71)	21.3% (4.06 - 58.14)	21.44% (4.17 - 59.08)	21.1% (3.9 - 59.26)
Cote d'Ivoire	17.84% (3.74 - 22.75)	21.55% (3.55 - 44.35)	23.46% (4.22 - 52.42)	23.25% (4.05 - 52.9)	23.53% (4.71 - 52.85)	24.38% (5.4 - 55.18)	24.77% (8.67 - 45.43)	22.4% (4.73 - 51.07)	21.56% (4.12 - 49.88)	20.81% (3.97 - 47.9)	19.59% (5.28 - 34.77)	20.23% (3.98 - 47.25)	20.38% (3.72 - 49.76)	20.17% (3.65 - 50.44)	19.71% (3.55 - 50.18)	18.86% (3.34 - 47.03)	18.6% (3.23 - 46.97)
DRC	37.44% (10.89 - 74.52)	36.33% (16.63 - 60.32)	40.58% (15.09 - 78.72)	41.53% (15.98 - 80.9)	37.99% (20.77 - 64.23)	39.85% (14.85 - 79.13)	42.71% (18.45 - 83.45)	46.95% (36.71 - 79.84)	42.5% (19.64 - 83.92)	42.24% (18.27 - 85)	42.17% (19.22 - 84.05)	44.84% (35.72 - 78.69)	42.22% (20 - 85)	40.66% (16.16 - 85.42)	39.32% (15.36 - 84.46)	37.43% (13.89 - 82.24)	36.64% (13.37 - 81.49)
Djibouti	37.11% (12.91 - 75.92)	35.31% (9.12 - 78.75)	34.47% (8.31 - 79.11)	33.49% (7.72 - 77.95)	32.97% (7.68 - 77.75)	32.91% (7.72 - 77.85)	32.11% (7.14 - 76.87)	30.2% (6.11 - 74.42)	29.81% (6.41 - 73.43)	28.81% (5.97 - 71.67)	29.02% (6.55 - 72.07)	30.05% (7.26 - 74.18)	30.11% (7.46 - 74.59)	30.05% (7.3 - 76.15)	29.4% (7.2 - 74.67)	29.51% (7.19 - 76.5)	27.81% (6.65 - 73.05)
Equatorial Guinea	32.7% (6.2 - 82.17)	34.15% (6.74 - 84.67)	34.72% (6.93 - 85.88)	33.3% (6.63 - 84.02)	33.57% (6.4 - 85.3)	33.52% (6.57 - 84.43)	33.5% (6.49 - 85.26)	34.29% (6.94 - 88.23)	31.94% (6.18 - 85.04)	32.83% (6.32 - 87.29)	33.16% (5.72 - 88.87)	34.23% (6.2 - 90.07)	34.9% (6.48 - 91.58)	35.47% (6.62 - 92.18)	35.55% (6.37 - 93.09)	35.35% (6.49 - 93.2)	34.82% (6.5 - 93.18)
Eritrea	42.45% (13.71 - 86.77)	43.28% (14.37 - 87.75)	43.24% (14.79 - 87.88)	39.91% (11.84 - 84.71)	39.39% (11.87 - 84.46)	37.16% (10.32 - 82.48)	36.65% (9.98 - 81.47)	35.25% (8.77 - 80.23)	33.99% (8.09 - 79.53)	31.04% (6.08 - 76.6)	29.7% (5.4 - 73.97)	28.37% (4.57 - 73.43)	27.98% (4.86 - 73.03)	27.34% (4.37 - 72.26)	26.87% (4.24 - 73.45)	26.65% (4.15 - 72.18)	26.06% (4.12 - 72.33)

Eswatini	28.06% (3.36 - 57.81)	27.67% (3.11 - 59.04)	28.54% (3.41 - 60.88)	28.43% (3.87 - 60.69)	28.8% (5 - 57.1)	28.99% (4.16 - 62.84)	28.35% (3.9 - 62.63)	27.9% (3.64 - 64.61)	27.38% (3.5 - 63.75)	25.85% (3.07 - 60.13)	25.54% (3.2 - 59.6)	25.31% (3.11 - 60.56)	25.53% (3.26 - 61.78)	25.37% (3.2 - 60.63)	25.11% (3.21 - 61.21)	25.19% (3.28 - 62.62)	24.65% (3.13 - 62.31)
Ethiopia	38.14% (9.06 - 85.11)	37.15% (8.59 - 84.86)	37.56% (8.94 - 85.17)	37.12% (8.72 - 84.79)	38.27% (9.76 - 86.29)	37.77% (9.47 - 85.6)	35.38% (8.75 - 83.34)	34.12% (8.07 - 82.02)	32.67% (7.47 - 79.84)	31.75% (7.05 - 79.07)	29.32% (5.99 - 74.24)	29.16% (6.18 - 72.87)	28.76% (6.25 - 74.14)	28.1% (5.9 - 73.48)	27.24% (5.51 - 71.59)	27.13% (5.57 - 71.28)	26.52% (5.26 - 71.81)
Gabon	35.58% (10.02 - 80.13)	36.23% (10.8 - 81.7)	35.1% (10.11 - 80.66)	34.47% (10.39 - 79.59)	33.66% (10.88 - 77.5)	32.76% (11.07 - 73.89)	31.24% (15.53 - 60.89)	30.57% (9.23 - 71.49)	31.51% (9.97 - 75.32)	31.21% (9.76 - 75.32)	28.86% (7.94 - 71.52)	28.9% (8.61 - 71.83)	29.32% (9.5 - 71.22)	29.56% (16.02 - 61.4)	28.71% (9.84 - 70.81)	28.58% (8.85 - 74.01)	28.01% (8.13 - 73.98)
Gambia	16.13% (2.2 - 38.57)	16.45% (2.27 - 39.63)	15.38% (2.1 - 36.13)	13.8% (1.73 - 29.95)	10.72% (1.5 - 14.15)	14.3% (2.03 - 31.69)	16.17% (2.8 - 39.48)	18.59% (5.34 - 40.75)	17.85% (3.5 - 48.49)	17.46% (3.17 - 48.05)	17% (2.95 - 46.72)	14.32% (2.3 - 36.71)	14.17% (3.16 - 28.95)	13.54% (2.73 - 28.53)	14.23% (2.27 - 38.21)	14.55% (2.36 - 40.75)	14.51% (2.41 - 42.37)
Ghana	22.79% (6.44 - 35.31)	29.86% (8.84 - 63.9)	38% (22.76 - 71.64)	31.6% (10.73 - 69.71)	29.64% (11.34 - 59.92)	30.8% (15.72 - 56.7)	29.1% (8.68 - 65.71)	28.78% (9.02 - 66.66)	28.81% (13.57 - 55.48)	27.07% (8.27 - 62.31)	24.04% (9.18 - 44.4)	23.79% (9.77 - 42.11)	25.05% (7.14 - 57.51)	24.68% (10.32 - 47.64)	25.03% (6.75 - 61.45)	25.35% (6.9 - 63.67)	24.97% (6.38 - 64.45)
Guinea	33.6% (8.85 - 68.81)	34.17% (9.33 - 70.96)	34.07% (9.33 - 71.11)	31.58% (7.72 - 66.98)	31.28% (8.28 - 66.89)	32.71% (10.47 - 68.56)	35.03% (21.11 - 59.18)	31.39% (9.68 - 66.29)	29.28% (8.04 - 61.44)	27.32% (6.95 - 56.81)	21.55% (6.16 - 31.45)	26.27% (7.04 - 53.99)	29.52% (15.04 - 51.42)	26.93% (7.52 - 57.71)	26.38% (7.17 - 58.45)	28.64% (13.01 - 54.04)	25.41% (6.67 - 56.79)
Guinea-Bissau	31.4% (15.67 - 59.85)	29.39% (8.91 - 67.16)	28.34% (7.96 - 66.98)	27.24% (7.01 - 66.23)	25.72% (5.98 - 63.65)	23.91% (5.1 - 58.49)	24.29% (5.59 - 59.58)	23.76% (5.79 - 56.81)	20.66% (6.52 - 37.84)	21.95% (5.09 - 53.87)	21.22% (4.45 - 52.86)	20.73% (4.53 - 50.86)	18.51% (5.74 - 32.46)	19.57% (4.19 - 49.62)	19.63% (4.08 - 51.03)	19.94% (4.05 - 54.5)	19.63% (3.8 - 53.66)
Kenya	35.96% (10.01 - 73.67)	38.05% (13.28 - 76.27)	43.94% (29.07 - 76.4)	34.47% (13.35 - 64.53)	31.42% (8.63 - 64.41)	28.82% (10.63 - 47.89)	33.2% (10.7 - 68.67)	38.6% (22.82 - 70.69)	40.46% (28.34 - 71.99)	36.76% (20.98 - 67.93)	32.54% (10.46 - 71.01)	31.25% (8.84 - 71.07)	29.82% (7.54 - 69.32)	26.04% (5.84 - 58.49)	20.45% (4.65 - 35.68)	25.63% (6.14 - 59.45)	27.26% (6.67 - 64.71)
Liberia	36.02% (9.73 - 75.84)	36.87% (11.18 - 76.98)	40.43% (16.27 - 81.27)	42.56% (29.42 - 74.4)	40.12% (17.15 - 80.46)	36.25% (20.03 - 64.8)	32.7% (10.24 - 69.37)	25.3% (8.69 - 41.09)	33.14% (10.19 - 72.96)	37.77% (14.47 - 81.42)	43.36% (33.42 - 78.5)	36.27% (14.44 - 78.54)	33.89% (12.54 - 75.64)	31.5% (16.33 - 59.39)	30.65% (9.66 - 69.76)	30.53% (9.69 - 71.6)	30.1% (8.94 - 71.54)
Madagascar	28.03% (7.64 - 63.47)	31.52% (11.21 - 69.98)	37.96% (26.43 - 71.95)	32.32% (12.4 - 72)	29.4% (9.76 - 65.77)	22.42% (7.32 - 40.77)	16.89% (3.71 - 26.12)	29.2% (13.97 - 59.89)	30.59% (11.56 - 71.81)	32.31% (13.9 - 74.91)	34.94% (23.43 - 72.35)	30.28% (13 - 70.39)	26.37% (13.63 - 52.53)	26.75% (9.35 - 65.25)	25.45% (8.82 - 62.82)	25.78% (13.94 - 52.8)	24.82% (8.53 - 62.29)
Malawi	15.53% (2.84 - 15.85)	20.5% (3.28 - 40.82)	23.24% (4.29 - 51.21)	25.16% (5.47 - 54.74)	31.31% (17.18 - 54.98)	25.24% (6.31 - 55.74)	24.85% (8.63 - 44.64)	20.1% (6.25 - 28.33)	21.04% (6.12 - 35.18)	18.73% (5.41 - 26.27)	21.45% (4.76 - 46.51)	24.19% (9.38 - 45.01)	22.57% (5.5 - 50.91)	21.68% (8.55 - 36.12)	20.13% (4.07 - 46.27)	20.24% (4.02 - 49.83)	19.97% (3.92 - 50.21)
Mali	30.86% (10.18 - 45.39)	35.24% (9.29 - 68.45)	37.47% (11.35 - 72.41)	37.39% (18.56 - 60.71)	37.53% (11.86 - 73.92)	36.41% (11.08 - 71.65)	33.63% (13.22 - 57.5)	35.82% (11.09 - 72.23)	37.32% (12.68 - 74.93)	41.89% (27.52 - 71.9)	38.58% (14.52 - 77.88)	39.42% (15.18 - 79.48)	44.39% (31.54 - 79.38)	37.95% (14.96 - 78.83)	36.01% (12.58 - 75.52)	35.91% (17.71 - 69.39)	33.12% (10.48 - 72.44)
Mauritania	41% (14.78 - 78.55)	45.84% (29.84 - 78.2)	41.91% (16.23 - 80.7)	39.65% (13.11 - 79.59)	38.71% (13.18 - 77.34)	40.76% (22.62 - 73.02)	35.86% (10.9 - 73.97)	33.1% (8.88 - 69.57)	31.51% (8.21 - 65.84)	27.59% (8.87 - 47.12)	30.87% (8.17 - 67.03)	32.18% (8.76 - 71.33)	32.77% (9.09 - 72.53)	32.9% (10.28 - 72.07)	33.23% (16.36 - 62.9)	30.74% (9.16 - 69.01)	29.96% (7.64 - 69.46)
Mozambique	18.69% (3.03 - 45.6)	16.33% (2.46 - 36.55)	10.89% (1.63 - 11.91)	16.61% (2.69 - 36.42)	21.03% (4.99 - 51.82)	23.73% (10.28 - 48.5)	22.35% (5.6 - 59.66)	21.12% (4.77 - 57.39)	18.7% (3.48 - 52.54)	17.98% (3.29 - 48.44)	18.74% (3.91 - 51.36)	18.84% (4.28 - 49.8)	19.28% (7.5 - 39.78)	18.74% (4.55 - 50.1)	18.51% (4.17 - 52.45)	18.55% (4.12 - 53.67)	17.31% (3.66 - 50.98)
Namibia	27.89% (5.4 - 70.63)	28.76% (6.1 - 72.12)	28.59% (6.28 - 71.23)	27.55% (5.85 - 70.4)	27.38% (5.76 - 70.52)	26.47% (5.6 - 68.54)	25.05% (5.55 - 63.86)	21.47% (5.79 - 43.96)	22.71% (4.49 - 57.12)	23.67% (4.49 - 63.67)	23.79% (4.59 - 65.68)	24.3% (5.12 - 66.46)	24.55% (5.07 - 68.4)	24.52% (5.13 - 69.37)	24.2% (5.16 - 70.16)	24.08% (5.1 - 70.43)	23.59% (4.69 - 69.63)
Niger	25.61% (8 - 40.89)	29.24% (6.85 - 63.12)	29.23% (6.82 - 65.74)	27.37% (5.58 - 62.29)	26.1% (5.12 - 59.78)	25.02% (5.19 - 55.78)	23.23% (6.83 - 39.39)	24.08% (4.85 - 54.2)	24.28% (4.74 - 58.65)	23.36% (4.32 - 57.18)	23.71% (4.47 - 57.53)	23.77% (4.64 - 58.38)	23.87% (4.97 - 59.11)	24.35% (5.37 - 61.63)	25.55% (6.41 - 62.81)	30.48% (16.08 - 62.59)	25.3% (6.43 - 62.95)

Nigeria	33.88% (7.59 - 61.7)	29.89% (10 - 38.42)	42.76% (26.26 - 65.67)	38.94% (13.55 - 71.27)	40.34% (23.29 - 62.15)	31.4% (13.08 - 42.48)	30.08% (6.91 - 54.73)	26.51% (8.55 - 33.74)	29.33% (7.03 - 54.96)	29.98% (11.99 - 42.44)	31.37% (14.53 - 45.06)	28.73% (7.04 - 54.51)	24.94% (8.28 - 32.17)	28.17% (6.4 - 56.1)	29.07% (6.95 - 58.99)	29.49% (12.08 - 46.63)	28.78% (6.98 - 59.77)
Republic Of Congo	38.18% (11.7 - 84.5)	36.67% (10.18 - 83.44)	37.5% (11.37 - 83.49)	37.9% (12.6 - 83.77)	36.57% (12.6 - 80.86)	34% (17.67 - 66.03)	35.34% (13.13 - 79.17)	35.51% (14.01 - 78.75)	34.87% (21.58 - 67.01)	36.04% (14.9 - 81.29)	33.47% (11.35 - 79.37)	34.64% (12.38 - 81.51)	35.54% (13.23 - 83.35)	35.01% (12.86 - 83.27)	34.51% (12.82 - 83)	35.08% (13.51 - 84.18)	34.12% (12.67 - 83.8)
Rwanda	28.72% (4.07 - 55.76)	28.91% (4.61 - 56.48)	28.09% (4.18 - 55.13)	27.85% (4.51 - 53.58)	28% (7.01 - 43.52)	28.65% (5.32 - 54.23)	29.38% (5.85 - 58.13)	31.5% (10 - 53.3)	29.62% (6.43 - 58.45)	29.96% (9.62 - 49.67)	25.65% (4.62 - 49.44)	24.03% (5.59 - 37.73)	25.61% (4.85 - 50.83)	27.04% (7.74 - 46.31)	25.66% (4.61 - 54.27)	25.43% (4.23 - 55.58)	25.04% (4.28 - 56.22)
Sao Tome And Principe	25.19% (5.11 - 60.7)	24.57% (5.32 - 58.85)	24.05% (6.22 - 52.45)	23.03% (4.69 - 55.31)	23.13% (4.68 - 57.08)	23.28% (4.86 - 58.6)	22.24% (4.46 - 56.36)	22.71% (4.94 - 56.23)	21.64% (5.27 - 49.45)	22.43% (4.9 - 57.74)	21.7% (4.24 - 57.44)	20.4% (3.84 - 53.5)	19.81% (3.67 - 51.76)	17.75% (3.49 - 41.96)	19.49% (3.79 - 51.58)	20.29% (4.04 - 56.86)	20.22% (3.94 - 57.96)
Senegal	48.68% (25.66 - 73.18)	43.29% (12.92 - 72.75)	44% (20.18 - 66.52)	34.97% (7.07 - 59.21)	28.77% (6.08 - 36.96)	32.32% (5.73 - 56.22)	36.95% (11.38 - 58.8)	30.56% (5.16 - 52.75)	24.27% (3.69 - 32.32)	26.66% (5.1 - 37.27)	26.4% (4.66 - 38.53)	38.13% (14.51 - 61.82)	38.99% (14.24 - 66.72)	34.45% (9.5 - 60.81)	30.31% (5.89 - 55.65)	28.96% (4.55 - 55.58)	28.08% (4.59 - 56.34)
Sierra Leone	22.59% (3.75 - 47.53)	24.91% (5.3 - 53.21)	29.66% (12.94 - 53.69)	20.79% (3.68 - 41.98)	15.62% (3.23 - 17.9)	17.86% (2.6 - 34.59)	18.62% (3 - 37.69)	18.97% (5.29 - 26.78)	17.47% (2.5 - 36.58)	17.82% (2.72 - 38.51)	17.39% (4.43 - 25.51)	14.15% (2.9 - 17.69)	17.88% (3.2 - 36.92)	22.42% (8.79 - 40.3)	19.59% (3.96 - 45.12)	18.74% (3.45 - 46.39)	18.21% (3.24 - 46.04)
Somalia	54.8% (43.4 - 87.72)	52.69% (29.48 - 90.85)	51.48% (27.08 - 90.68)	50.65% (25.53 - 90.46)	52.39% (30.51 - 91.53)	53.4% (43.73 - 88.01)	52.86% (30.78 - 92.52)	51.72% (27.1 - 92.59)	50.6% (24.95 - 92.58)	50% (24.73 - 92.3)	49.23% (23.91 - 92.16)	51.65% (27.14 - 94.16)	50.14% (25.07 - 93.5)	49.99% (25.26 - 93.36)	49.48% (24.06 - 93.49)	49.52% (24.32 - 93.87)	48.85% (23.79 - 93.66)
South Africa	31.78% (5.61 - 78.64)	32.46% (6.05 - 79.58)	32.71% (6.72 - 80.33)	31.71% (6.02 - 80.21)	29.95% (5.34 - 77.88)	29.81% (5.3 - 77.89)	29.46% (5.15 - 77.52)	29.29% (5.23 - 78.61)	28.95% (5.07 - 77.91)	28.42% (4.86 - 77.74)	27.93% (4.72 - 77.63)	27.83% (4.79 - 77.99)	27.53% (4.64 - 77.97)	25.99% (4.19 - 75.65)	25.68% (4.19 - 74.65)	25.68% (4.42 - 76.25)	25.18% (4.03 - 75.07)
South Sudan	38.26% (13.06 - 82.25)	39.46% (13.94 - 84.2)	40.14% (15.38 - 84.42)	39.75% (16.71 - 83.03)	40.74% (31.79 - 72.37)	37.29% (15.82 - 79.29)	33.87% (9.73 - 79.17)	32.58% (8.75 - 78.81)	32.01% (8.76 - 77.7)	30.77% (7.32 - 76.93)	32.97% (10.53 - 78.64)	31.75% (8.79 - 79.52)	31.93% (8.97 - 79.15)	30.6% (7.9 - 77.94)	30.45% (8.34 - 77.79)	30.59% (8.6 - 78.22)	30.28% (8.36 - 78.14)
Sudan	37.79% (14.86 - 86.65)	38.32% (16.05 - 87.01)	37.24% (15.01 - 85.42)	34.59% (15.34 - 81.74)	31.21% (19.36 - 64.35)	31.63% (12.35 - 76.71)	33.07% (12.71 - 81.74)	33.71% (13.57 - 83.35)	33.56% (13.31 - 83.9)	33.47% (12.97 - 84.8)	33.02% (12.46 - 83.85)	33.07% (12.82 - 84.73)	33.14% (12.5 - 85.48)	32.74% (12.46 - 85.62)	31.3% (12.1 - 83.72)	31.48% (12.06 - 83.64)	31.11% (11.4 - 84.11)
Tanzania	34.41% (11.39 - 72.78)	37.03% (22.06 - 66.77)	33.89% (11.39 - 72.19)	31.94% (9.97 - 69.34)	30.64% (14.92 - 54.27)	33.04% (11.65 - 71.79)	34.46% (20.71 - 64.36)	31.2% (10.14 - 69.53)	30.15% (9.93 - 67.63)	30.23% (15.89 - 56.58)	27.57% (8.34 - 62.53)	23.73% (8.77 - 42.3)	27.61% (8.09 - 65.45)	28.81% (8.76 - 70.47)	27.79% (8.05 - 68.13)	28.12% (8.65 - 69.92)	27.97% (8.2 - 69.96)
Togo	15.98% (4.97 - 26.71)	21.2% (6.27 - 55.14)	23.11% (7.41 - 61.18)	23.41% (8.32 - 61.42)	23.83% (14.61 - 49.39)	22.92% (8.28 - 59.65)	22.18% (7.89 - 59.51)	25.36% (16.69 - 55.9)	22.98% (8.75 - 62.65)	22.71% (8.22 - 64.72)	23.27% (9.62 - 64.79)	24.84% (18.24 - 54.95)	24.76% (11.05 - 69.52)	24.27% (9.34 - 72.01)	23.3% (8.58 - 70.23)	21.8% (8.06 - 66.14)	20.96% (7.46 - 64.58)
Uganda	48.37% (38.24 - 80.3)	39.14% (14.03 - 77.96)	35.36% (10.58 - 71.03)	34.42% (16.52 - 59.09)	32.23% (9.17 - 66.19)	31.73% (14.77 - 52.52)	30.26% (7.83 - 64.07)	27.92% (6.63 - 58.92)	25.4% (8.53 - 41.01)	27.24% (6.76 - 57.78)	26.45% (10.51 - 42.37)	27.18% (6.99 - 58.66)	25.01% (8.94 - 41.74)	27.18% (6.73 - 62.12)	27.83% (6.66 - 66.77)	27.06% (6.22 - 64.48)	26.93% (6.29 - 65.22)
Zambia	38.81% (12.39 - 76.78)	44.23% (28.54 - 76.06)	40.73% (15.3 - 80.21)	40.19% (14.18 - 81.19)	39.21% (13.76 - 80.47)	39.7% (14.36 - 80.81)	41.37% (17.5 - 82.75)	43.63% (31.59 - 76.58)	39.41% (16.44 - 80.6)	37.81% (13.61 - 79.91)	36.75% (12.79 - 79.34)	35.22% (12.88 - 75.19)	32.9% (16.29 - 61.53)	35.91% (12.98 - 78.14)	36.56% (13.19 - 80.82)	37% (13.95 - 81.4)	35.44% (12.51 - 79.92)
Zimbabwe	35.61% (9.89 - 76.01)	37.47% (11.75 - 78.74)	39.9% (14.87 - 81.07)	37.03% (18.36 - 68.61)	31.44% (10.42 - 61.73)	32.82% (9.02 - 71.29)	33.49% (9.81 - 74.07)	34.74% (11.68 - 75.59)	37.42% (20.09 - 72.76)	34.89% (14.44 - 71.86)	32.08% (9.54 - 72.45)	31.76% (9.57 - 72.82)	31.66% (9.46 - 72.03)	30.55% (11.22 - 65.37)	30.87% (9.03 - 73.65)	31.12% (8.92 - 75.24)	29.44% (8.18 - 71.86)
