# Supplementary material for: Estimating rates of treatment delay for malaria fevers among children in Sub-Saharan Africa 2006–2022
Source: Nat Commun. 2025 Oct 29;16:9534. doi: 10.1038/s41467-025-64584-8 (PMC12572339; doi:10.1038/s41467-025-64584-8)
Supplement: Supplementary file 2 — Description of Additional Supplementary Files [file 41467_2025_64584_MOESM2_ESM.pdf]

Description of Additional Supplementary Files

Supplementary Data 1: Country population-weighted delayed treatment proportion between 2006 and 2022 by over 24 hours
