# Supplementary material for: Estimating rates of treatment delay for malaria fevers among children in Sub-Saharan Africa 2006–2022
Source: Nat Commun. 2025 Oct 29;16:9534. doi: 10.1038/s41467-025-64584-8 (PMC12572339; doi:10.1038/s41467-025-64584-8)
Supplement: Supplementary file 4 — Reporting Summary [file 41467_2025_64584_MOESM4_ESM.pdf]

nature portfolio

Corresponding author(s): Jailos Lubinda

Last updated by author(s): August 20, 2025

Reporting Summary

Nature Portfolio wishes to improve the reproducibility of the work that we publish. This form provides structure for consistency and transparency in reporting. For further information on Nature Portfolio policies, see our Editorial Policies and the Editorial Policy Checklist.

Statistics

For all statistical analyses, confirm that the following items are present in the figure legend, table legend, main text, or Methods section.

n/a	Confirmed	
☐	☒	The exact sample size (*n*) for each experimental group/condition, given as a discrete number and unit of measurement
☐	☒	A statement on whether measurements were taken from distinct samples or whether the same sample was measured repeatedly
☒	☐	The statistical test(s) used AND whether they are one- or two-sided<br>*Only common tests should be described solely by name; describe more complex techniques in the Methods section.*
☐	☒	A description of all covariates tested
☐	☒	A description of any assumptions or corrections, such as tests of normality and adjustment for multiple comparisons
☐	☒	A full description of the statistical parameters including central tendency (e.g. means) or other basic estimates (e.g. regression coefficient) AND variation (e.g. standard deviation) or associated estimates of uncertainty (e.g. confidence intervals)
☐	☒	For null hypothesis testing, the test statistic (e.g. *F*, *t*, *r*) with confidence intervals, effect sizes, degrees of freedom and *P* value noted<br>*Give P values as exact values whenever suitable.*
☐	☒	For Bayesian analysis, information on the choice of priors and Markov chain Monte Carlo settings
☒	☐	For hierarchical and complex designs, identification of the appropriate level for tests and full reporting of outcomes
☒	☐	Estimates of effect sizes (e.g. Cohen's *d*, Pearson's *r*), indicating how they were calculated

Our web collection on statistics for biologists contains articles on many of the points above.

Software and code

Policy information about availability of computer code

Data collection	All the data used in this study are those collected by the Demographic Health Surveys Program (DHS), and the Unicef Multiple Indicator Cluster Survey (MICS). We did not collect the survey data from the field, but do extract it using customized code built into an in-house data extraction pipeline using PostgreSQL RDBMS.
Data analysis	We used R programme (v. 4.1) for model implementation, data preprocessing, and result visualization. We also used ArcGIS v10.8 for creating of maps.

For manuscripts utilizing custom algorithms or software that are central to the research but not yet described in published literature, software must be made available to editors and reviewers. We strongly encourage code deposition in a community repository (e.g. GitHub). See the Nature Portfolio guidelines for submitting code & software for further information.

Data

Policy information about availability of data

All manuscripts must include a data availability statement. This statement should provide the following information, where applicable:

- Accession codes, unique identifiers, or web links for publicly available datasets
- A description of any restrictions on data availability
- For clinical datasets or third party data, please ensure that the statement adheres to our policy

- A code framework is shared through Gitlab https://github.com/Jailos/treatment-delay

All data supporting the findings of this study are available within the paper and its Supplementary Information. Detailed, unaggregated data are publicly accessible through the Demographic and Health Surveys (DHS) Programme (https://dhsprogram.com/) and the UNICEF Multiple Indicator Cluster Surveys (MICS) (https://mics.unicef.org/) websites. Access to DHS and MICS datasets requires free registration and approval from the respective data providers. The geographic shapefiles used to generate mapped results are available for download through the Malaria Atlas Project R package (https://cran.r-project.org/web/packages/malariaAtlas/index.html).

Research involving human participants, their data, or biological material

Policy information about studies with human participants or human data. See also policy information about sex, gender (identity/presentation), and sexual orientation and race, ethnicity and racism.

Reporting on sex and gender	The results reported here are all for both sexes. While we explored sex-based analysis, and any differences between them. We do not report the full results here as the differences were not significant. Based on DHS protocols all sex classification was based on self-reported, and as assigned at birth. The data was comprised of 48.8% female and 51.2% males. The models were run on de-anonymised aggregated data, totalling 11,325 individual-level records.
Reporting on race, ethnicity, or other socially relevant groupings	We used socio-economic categorisations using wealth quintiles ranked into five equal parts, from quintile one (lowest-poorest) to quintile five (highest-wealthiest) as an indicator of the economic status of households. These are detailed information on dwelling, household characteristics and access to a variety of consumer goods and services, and assets, which together are used as a measure of economic status to indicate inequalities in the use of health and other services, and in health outcomes.<br><br>We also analysed the data based on rural and urban categorisations, which are area-specific indicators. This distinction highlights how population density affects the distribution of accessibility to health infrastructure, costs of access, and physical distance to health facilities, and thereby having potential influences on delay estimates and subsequently, overall health outcomes between people living in rural and urban areas.<br><br>For details of how these categorizations are derived, we offer references that address the methodology at greater depths, particularly: Rutstein, S. O. and S. Staveteig. 2014. Making the Demographic and Health Surveys Wealth Index comparable. DHS Methodological Reports No. 9. Rockville, Maryland, USA: ICF International. https://dhsprogram.com/publications/publication-mr9-methodological-reports.cfm
Population characteristics	We describe the covariates used in detail in the manuscript, all being related to access to health systems, health systems performance, and population level indices. All covariates used are obtained from well documented sources including the Institute of Health Metrics Evaluation (IHME), and the Malaria Atlas Project. The participant data used in this study is based on children aged between 0 and 59 months (referred to as under 5-year-olds).
Recruitment	As we are a third-party user of the survey data, we briefly describe the inclusion and exclusion criteria, but do not offer explanation of survey specific sampling frame or biases thereof, which we are confident have all been taken care of by the DHS program and UNICEF during the survey design and data collection stages. Nonetheless, we clearly explain that we included all children that had a fever in the 2 weeks preceding the survey, who took an antimalarial for treatment, and gave information about how long since symptom onset it had been at the point of taking the antimalarial treatment.
Ethics oversight	As we did not do the surveys, we did not have to seek ethical approval from every country. Rather, the DHS and UNICEF MICS programs do obtain ethics oversight approvals from the countries of surveys. We have permission for use of the data for research purposes, as third parties and this is publicly available.

Note that full information on the approval of the study protocol must also be provided in the manuscript.

Field-specific reporting

Please select the one below that is the best fit for your research. If you are not sure, read the appropriate sections before making your selection.

☒ Life sciences ☐ Behavioural & social sciences ☐ Ecological, evolutionary & environmental sciences

For a reference copy of the document with all sections, see nature.com/documents/nr-reporting-summary-flat.pdf

Life sciences study design

All studies must disclose on these points even when the disclosure is negative.

Sample size	As this study is based on retrospective cross-sectional surveys, the survey sample based on DHS and MICS protocols are notionally representative. In Our case, we had access to 111 325 observations across 40 countries between 2006 and 2022.
Data exclusions	Outlier data were excluded at survey level. The excluded surveys comprised four MICS and two DHS, which were excluded for having too small samples or for having estimates inconsistent with other survey results conducted within the same country. The surveys excluded comprise one for Djibouti in 2006, one for Rwanda in 2008, another for Senegal in 2020 and two in Eswatini for 2010 and 2014, all of which had sample sizes falling below the threshold of 25 and insufficient for inclusion. Furthermore, a fourth MICS survey conducted in Mozambique in 2008 was excluded due to inconsistencies in the survey results compared to other surveys conducted within the same country
Replication	To ensure reproducibility, models were run in R independently multiple times to assess the stability of the results, and internal INLA

Replication	diagnostics and validation metrics such as the Probability Conditional Ordinates (PCO) and the Predictive Integral Transform (PIT) were collected. The mean of the log of the CPO values for our model indicates generally good and stable predictive performance. We also did a traditional 10 fold cross-validation and assess both the in-sample and out-of-sample performance of the model.
Randomization	Based on DHS and MICS survey protocols and study designs, usually two-stage probability samples are drawn from an existing sample frame, generally the most recent census frame, using probability sampling of units (selected randomly with known and nonzero probabilities). The stratification helps reduce sampling errors, depending on the population variance existing within the strata but not between the strata. Typically, DHS samples are stratified by geographic region and by urban/rural areas within each region. Within each stratum, the sample design specifies an allocation of households to be selected.
Blinding	This was not applicable to this study

Reporting for specific materials, systems and methods

We require information from authors about some types of materials, experimental systems and methods used in many studies. Here, indicate whether each material, system or method listed is relevant to your study. If you are not sure if a list item applies to your research, read the appropriate section before selecting a response.

Materials & experimental systems

n/a	Involved in the study
☒	☐ Antibodies
☒	☐ Eukaryotic cell lines
☒	☐ Palaeontology and archaeology
☒	☐ Animals and other organisms
☒	☐ Clinical data
☒	☐ Dual use research of concern
☒	☐ Plants

Methods

n/a	Involved in the study
☒	☐ ChIP-seq
☒	☐ Flow cytometry
☒	☐ MRI-based neuroimaging

Plants

Seed stocks	We did not use any plant information in this study
Novel plant genotypes	We did not use any plant information in this study
Authentication	We did not use any plant information in this study
